# The knowledge, attitudes and practices of hand, foot, and mouth disease prevention strategies amongst parents and educators of children under 5 years amidst COVID-19 pandemic: A cross-sectional study

**DOI:** 10.3389/fpubh.2022.908004

**Published:** 2022-10-17

**Authors:** Min Xian Wang, Junxiong Pang

**Affiliations:** ^1^Saw Swee Hock School of Public Health, National University of Singapore and National University Health System, Singapore, Singapore; ^2^Centre for Infectious Disease Epidemiology and Research, National University of Singapore, Singapore, Singapore

**Keywords:** knowledge attitude and practises, parents, teacher, hand foot and mouth disease (HFMD), prevention strategies

## Abstract

**Background:**

Hand, foot, and mouth disease (HFMD) is endemic in Singapore. Prevention efforts have been ramped up since major outbreaks in the early 2000's. This study aims to assess the current knowledge, and attitudes towards and practise (KAP) levels of HFMD prevention strategies (HFMD-PS) amongst parents and teachers of children under 5 years amidst the COVID-19 pandemic.

**Methods and results:**

A convenience sample of 240 teachers and 404 parents responded to a self-administered standardised questionnaire between mid-October and December 2020. A scoring framework was used to assess responses in the ‘knowledge', ‘attitude', and ‘practice' domains. A multivariable analysis was adjusted for ethnicity and attitudes towards getting children to follow proper handwashing steps and regularly disinfecting children's toys amongst parents, knowledge about HFMD's infectious period, and the responses to a child turning symptomatic in the childcare centre amongst teachers. Existing levels of knowledge and attitudes of parents and teachers were not high, and only a small proportion practised high levels of prevention measures (99 parents and 28 teachers). Key facilitators for a higher practise level in parents include the following: (1) awareness of regular liquid soap's efficacy as a disinfectant, (2) toy cleaning before and after playtime, and (3) the cleaning agent used for this practise. Teachers had no significant factors associated with higher practise levels.

**Conclusion:**

This study suggested potential gaps between positive knowledge and attitudes towards prevention strategies and their actual adoption levels in homes and childcare centres during COVID-19 pandemic. These evidences suggest the importance of continuous promotion of HFMD prevention practise in homes and childcare centres, even amidst pandemics.

## Introduction

Hand, foot, and mouth disease (HFMD) has been endemic in Singapore, since the first case was reported in 1970. The clinical features and virology of HFMD have been well-explored in the literature, and its epidemiological landscape in Singapore has been succinctly summarised in this study by Kua et al. (1). Local health authorities had increased efforts to prevent transmission through various approaches. These include, but are not limited to, establishing a set of infection control guidelines in high-risk environments such as childcare centres and indoor playgrounds and increased advocacy for HFMD-related knowledge and prevention strategies to children's main caregivers. Despite elevated efforts, HFMD still poses a notable burden on Singapore's economy and healthcare system, with HFMD-related medical costs and productivity losses in 2016 estimated at $4.8 million (2).

HFMD was a notifiable disease in Singapore from 2000 until 2019. With the absence of an effective HFMD vaccine, the current HFMD prevention strategies (HFMD-PS) against infection are mainly focused on practising high levels of personal and environmental hygiene. The weekly incidence of HFMD cases was notably lower in 2020 than in 2019, but gradually increased in 2021, though not to pre-pandemic levels in 2019 (3–5). Reduced levels in 2020 can be attributed to the nationwide COVID-19 circuit breaker measures, as the weekly incidence levels have been decreasing from March (epidemiological week 9) but only maintained at the minimum from April 2020 (epidemiological week 14). This coincides with a gradual introduction of COVID-19-related restrictions in Singapore such as improved hygiene standards in pre-schools from 14 February 2020 (6), leading to childcare centre closures when the circuit breaker was imposed on 8 April 2020 (7). These real-world evidences support the high efficacy of good personal and environmental hygiene practises in preventing HFMD, which is also generally observed in the literature (1, 8).

Given the importance of good personal and environmental hygiene practises for HFMD prevention, it is important that the main caregivers of the most susceptible group—children aged under 5 years—have accurate knowledge and receptive attitudes towards HFMD and its prevention strategies. However, there are mixed observations on the plausible association between knowledge and attitudes towards actual practise of HFMD-PS from studies in Malaysia, Thailand, and China (as further discussed in later sections) (8–12). Within Singapore, an exploration of the knowledge, and attitudes towards and practise (KAP) of good hand hygiene practises for preventing gastrointestinal diseases, another infectious disease with similar transmission pathways to HFMD, found a gap between having good knowledge and attitudes towards good hand hygiene practises, to actual practise in reality (13). Nonetheless, assessments of KAP of HFMD and HFMD-PS in parents and early childhood educators in Singapore are still limited, especially amidst the COVID-19 pandemic.

The primary objective of this study was to assess the current knowledge, and attitudes towards and practise (KAP) levels of HFMD prevention strategies (HFMD-PS) amongst parents and pre-school teachers during the COVID-19 pandemic. The secondary objective of this study was to identify facilitators and barriers to good practise of HFMD-PS amongst childcare teachers and parents. The study findings may assist in guiding future policies and strategies to encourage good practise of HFMD-PS amongst the caregivers.

## Methods

### Participants and data collection

Respondents of this cross-sectional study were recruited from early childhood educators (referred to as teachers from this point on) employed across 35 childcare centres located in various parts of Singapore and parents of children attending these childcare centres. Data were collected from the respondents between mid-October and December 2020 through self-administered structured questionnaire standardised for teachers and parents. The respective questionnaires were disseminated through electronic means—email blasts—to all teachers and parents through the principals of each childcare centre. The questionnaires were hosted on RedCap, a secure electronic platform. All respondents gave informed consent prior to questionnaire commencement.

This study was approved by the National University of Singapore Institutional Review Board (NUS-IRB S-19-132, approved on 3 May 2019) prior to data collection, and written informed consent was waived for all respondents.

### Data management and scoring framework

All questionnaire records were extracted from RedCap for analysis by a member of the research team involved in data collection. Data cleaning and analysis was subsequently performed by a researcher independent from the original team involved in data collection. Each record was managed as a unique response and checked for completeness, accuracy, and clarity by the independent researcher before inclusion for analysis. Completeness of records is defined by having at least 95% completion rate for demographics and compulsory questions, before inclusion in analysis.

Each record included for analysis was scored based on a scoring system that grouped questions and sub-questions into ‘knowledge', ‘attitudes', and ‘practice' domains. An additional ‘experience' domain was also used to group questions that qualitatively explored the teachers' experience of HFMD-related events in their centre. The number of questions (including sub-questions) in each domain is as follows: knowledge (parents six, teachers six), attitude (parents eight, teachers six), practises (parents eight, teachers four), and experience (teachers six). A detailed breakdown of specific questions belonging to each domain and the exact scoring framework for parents and teachers are presented in Appendix A.

The broad scoring framework is as follows: all questions have a maximum score of 1 mark for providing positive responses and a minimum score of−1 mark when non-positive responses were provided, except for questions 12 and 15 for the educators where a minimum score of 0 was given for non-positive responses. For both teachers and parents, positive responses for knowledge and attitudes are defined as those reflecting accurate knowledge about HFMD and positive attitudes towards implementing strategies for HFMD prevention (either existing or future). Positive responses for practise are tiered into ‘best' and ‘good' practises. For teachers, a ‘best' practise is defined as a practise in line with regulatory guidelines by the Ministry of Health, Singapore, to prevent and control infectious disease transmission within pre-schools and a ‘good' practise is defined as a practise not recommended by the regulatory guidelines but may contribute to limiting infectious disease transmission within pre-schools (14). For parents, a ‘best' practise is defined by a practise that removes pathogens effectively from their child's living environment or supports the centre in preventing and controlling infectious disease transmission. Non-positive responses for practises are defined by a ‘poor' or ‘worse-than-poor' practise for educators and parents. A ‘poor' or ‘worse-than-poor' practise is defined by practises that do not contribute to preventing and controlling HFMD transmission within pre-schools or a child's living environment. Questions allowing multiple responses utilised additive scoring, where the average score of the positive responses given and that of the non-positive responses given were summed to give the question's total score. Questions providing responses for varying frequency or efficacy of a certain practise utilised ranked scoring, where marks increased proportionately with the frequency or efficacy of performing a certain practise. For these question types, the maximum and minimum marks were normalised to 1 mark and−1 mark, respectively. Finally, domain scores for Knowledge, attitudes and practices for teachers and parents were derived from the sum of the question scores for all questions grouped in each domain.

The range of scores for the respective domains is as follows: for parents—knowledge (−6 to 6), attitude (−8 to 8), and practise (−8 to 8); for teachers—knowledge (−6 to 6), attitude (−6 to 6), and practise (−9 to 9). For the purpose of facilitating interpretation and discussion, the authors defined the following in this study:

1. ‘Good' knowledge, attitudes, or practises: A positive domain score was interpreted as an overall good knowledge/attitude/practise of HFMD-PS.
2. ‘Poor' knowledge, attitudes, or practises: A negative domain score was interpreted as poor knowledge/attitude/practise of HFMD-PS.
3. ‘High' knowledge or attitude: The 75th percentile of the *maximum* domain scores were used as the respective cut-off scores determining ‘high' knowledge or attitude in each sub-population:
  a. Knowledge: a domain score of >4.5 for both parents and teachers,
  b. Attitude: a domain score of >6 for parents and >6 for teachers.
4. ‘High' practise: The 75th percentile of the *observed* practise scores were used as the respective cut-off scores determining ‘high' practise in each sub-population in this study: a domain score >4.35 for parents and >6.42 for teachers.

### Statistical analysis

Categorical variables were summarised with frequency and percentages, and continuous data were summarised with means ± standard deviations (SD). Differences between ‘high' and ‘low' practise groups were assessed using the Fisher's exact and Chi-squared tests for categorical variables and the Mann–Whitney and Kruskal–Wallis tests for continuous variables. Crude and adjusted associations for the likelihood of ‘high/low' practise with all variables and questions were explored with uni-variable and multivariable logistic regression models. Confounders were chosen using the stepwise command and likelihood ratio tests through two separate approaches in automatically and manually built multivariable models. The first approach added all variables, and the second approach included only variables that were significantly different between the high and low practise groups, based on the Fisher's exact test and uni-variable logistic regression results. Automatically and manually built models were compared and chosen based on their performance in receiver operating characteristics curve analysis and likelihood ratio test result. Confounders included in the final multivariable logistic regression model include ethnicity, Q16 and Q17 scores for parents, and Q6 and Q20 scores for teachers. A further adjustment of HFMD experience with the children or centre (demographic question for parents and Q22 for teachers) was also explored as an additional analysis on epidemiological grounds. All tests were performed with Stata 13 (StataCorp LP, College Station, TX, USA) and were two-sided with a significance value of *p* < 0.01 and 99% confidence intervals reported when applicable.

## Results

### Respondent demographic characteristics

#### Overall

A total of 644 of the 666 respondents were included in the analysis; five parents and 17 teachers had provided incomplete records and were excluded from the analysis. Parents' age (*n* = 404) range was between 24 and 46 years (mean 35.4 ± 5.37), while teachers' age (*n* = 240) range was between 18 and 71 years albeit with a similar mean age (35.5 ± 11.8). Generally, high formal education levels were observed in all respondents−89.7% of the respondents had at least post-secondary education qualifications, of whom 47% had at least a university degree. Most respondents were females (89.0%) and were affiliated with private or autonomous childcare centres (64.6%), rather than with public childcare centres (65.4%). The ethnic distribution amongst parents and teacher populations were similar to Singapore's ethnic distribution, where Chinese (68.3%) and Malays (17.4%) were the major ethnic groups. However, other ethnicities (8.07%) had a relatively larger representation than the Indians (5.9%) in this study.

#### Parents

Amongst the 404 parents analysed, 99 parents were classified as the ‘high practice' parent (HPP) group and the remaining 305 parents with practise scores ≤ 4.35 were classified as the ‘low practice' parent (LPP) group (*n* = 305). The HPP and LPP groups had similar demographics (Table 1), except for ethnic distribution (*p* < 0.0001, Table 1). General trends in education levels and ethnic group distribution in the HPP and LPP groups agreed with those observed in the overall parent population. However, a significantly larger proportion of respondents in the LPP group were Chinese (83.6%, Table 1) compared with those in the HPP group (62.2%, Table 1). Likewise, the proportions of Indian and other ethnicity respondents in the HPP group were approximately two to three times higher than those in the LPP group (Table 1). In both HPP and LPP groups, respondents mostly resided in public housing (HPP 82.8%, LPP 79.7%, Table 1), had two children (HPP 48.5%, LPP 46.9%, Table 1), and had mean 5 years of parenting experience (HPP 5.01 ± 2.90 years, LPP 5.28 ± 3.34 years, Table 1). An experience with HFMD infection in at least one child was more prevalent in the HPP group (55.6%, Table 1) than in the LPP group (41.6%, Table 1). However, the difference was not statistically significant (Fisher's exact *p* = 0.02). The median age of their child's HFMD infection was similar in both groups, averaging at 2.74 ± 1.43 years in the HPP group and 2.67 ± 1.96 years in the LPP group, as with HFMD re-infection rates (HPP 11.32%, LPP 10.1%, Table 1).

**Table 1:** Demographics of parent respondents.

**Parents**		**Number of respondents (%)**			***p***	**Crude OR (99% Cl)**	**Adjusted OR^a^ (99% CI)**	**Adjusted OR^b^ (99% CI)**
**Variable**		**Overall (*N* = 404)**	**High practise (*N* = 99)**	**Low practise (*N* = 305)**				
Gender	Male	70 (17.3)	22 (22.22)	48 (15.74)	0.17	1	1	1
	Female	334 (82.7)	77 (77.78)	257 (84.26)		0.65 (0.31–1.37)	0.46 (0.19–1.08)ramyaI!!	0.48 (0.20–1.13)ramyaI!!
Age	Mean (SD)	35.40 (5.37)	35.52 (5.27)	35.36 (5.41)	0.80	1.01 (0.95-1.06)	1.04 (0.97–1.11)	1.05 (0.98–1.12)
	Median (IQR)	35 (32–39); Range: 24-46	35 (32–39); Range: 26-50	35 (32–39); Range: 5-49	0.58	NA		
Ethnicity	Chinese	315 (78.4)	61 (62.24)	254 (83.55)	< 0.01	1	NA	
	Malay	56 (13.9)	21 (21.43)	35 (11.51)		2.50 (1.12–5.56)^*^		
	Indian	10 (2.5)	4 (4.08)	6 (1.97)		2.78 (0.51–15.24)		
	Others	21 (5.2)	12 (12.24)	9 (2.96)		5.55 (1.68–18.32)^*^		
Highest educational qualification	Pre-Primary/Primary/Secondary	29 (7.2)	11 (11.11)	18 (5.9)	0.18	1	1	1
	Post-secondary (Non-tertiary)	22 (5.5)	6 (6.06)	16 (5.25)		0.61 (0.13-2.98)	0.22 (0.03–1.39)ramyaI!!	0.19 (0.03–1.24)ramyaI!!
	Diploma courses	122 (30.2)	33 (33.33)	89 (29.18)		0.61 (0.20–1.85)	0.43 (0.13–1.48)	0.38 (0.11–1.32)ramyaI!!
	University and above	231 (57.2)	49 (49.5)	182 (59.7)		0.44 (0.15–1.28)ramyaI!!	0.46 (0.14–1.53)	0.44 (0.13–1.43)
Housing type	Public (HDB)	325 (80.5)	82 (82.83)	243 (79.7)	0.32	1	1	1
	Private (Semi/Full)	71 (17.6)	17 (17.17)	54 (17.7)		0.93 (0.42–2.05)	1.16 (0.47–2.86)	1.19 (0.48–2.95)
	Not reported/Unclear	8 (2)	0 (0)	8 (2.6)		Omitted	Omitted	Omitted
Type of childcare	Public	158 (39.1)	31 (31.31)	127 (41.64)	0.08	1	1	1
	Non-public (Private/Autonomous)	246 (60.9)	68 (68.7)	178 (58.4)		1.57 (0.83–2.95)	1.91 (0.94–3.86)ramyaI!!	1.80 (0.88-3.68)ramyaI!!
Number of children	1	150 (37.1)	36 (36.36)	113 (37.05)	0.96	1	1	1
	2	191 (47.3)	48 (48.48)	143 (46.89)		1.05 (0.55–2.03)	1.04 (0.51–2.15)	1.19 (0.56–2.50)
	≥3	64 (15.84)	15 (15.15)	49 (16.07)		0.96 (0.39–2.38)	0.81 (0.30–2.18)	1.07 (0.38–3.05)
Age of oldest child (Years)	Mean (SD)	5.21 (3.24)	5.01 (2.90)	5.28 (3.34)	0.46	0.97 (0.89–1.07)	0.98 (0.88–1.08)	1.00 (0.90–1.12)
	Median (IQR)	5 (3–6); Range: 0.33–21	4 (3.08–6); Range: 0.75-16	5 (3–6); Range: 0.33–21	0.74	NA		
Child with HFMD history	Yes	222 (45)	55 (55.56)	127 (41.64)	0.02	1	1	NA
	No	182 (55)	44 (44.44)	178 (58.36)		1.75 (0.96–3.19)ramyaI!!	1.81 (0.94–3.53)ramyaI!!	
Median age of HFMD infection (years)	Mean (SD)	2.68 (1.87)	2.74 (1.43)	2.67 (1.96)	0.77	1.02 (0.82–1.28)	1.06 (0.83–1.36)	1.06 (0.83–1.36)
	Median (IQR)	2.5 (1.67–3); Range: 0.33-19	2.5 (2–3); Range: 0.33-7.5	2 (1.5–3.5); Range: 0.5-19	0.38	NA		
HFMD re-infection in same child	Yes	23 (10.36)	6 (11.32)	17 (10.06)	0.80	1	1	1
	No	199 (89.64)	47 (88.68)	152 (89.94)		0.88 (0.24–3.20)	1.52 (0.34–6.73)	1.56 (0.34–7.04)

OR, Odds ratio;

* p < 0.01; ramyaI!!0.01 ≤ p < 0.05; NA, Not Applicable; p, Fisher's exact test p-value (categorical variable) or t-test p-value (continuous variable).

a adjusted for ethnicity, Q16 Score, Q17 Score.

b adjusted for ethnicity, Q16 Score, Q17 Score, HFMD Status (Yes as reference group).

#### Teachers

Amongst 240 teachers analysed, 28 teachers were classified as ‘high practice' teachers (HPTs) and the remaining 212 teachers with practise scores ≤ 6.42 were classified as ‘low practice' teachers (LPTs). In general, teachers in both groups were comparable in age (mean age HPT 35.1 ± 10.7 years, LPT 35.5 ± 11.93 years, Table 2) and full-time teaching status (overall 95%, HPT 92.9%, LPT 95.3%, Table 2) with a staff-to-children ratio of 6 to 15 (HPT 64.3%, LPT 57.4%, Table 2). When compared against the LPTs, HPTs were relatively less -ducated (60.7% with at least a diploma certificate), with a longer working experience (mean experience 7.25 ± 8.97 years, Table 2), had children (57.1%, Table 2), and sent their children to childcare, if any (68.8%, Table 2). Nonetheless, the HPT and LPT groups were not significantly different across all demographic variables analysed (*p* > 0.01, Table 2).

**Table 2:** Demographics of teacher respondents.

**Parents**		**Number of respondents (%)**			***p***	**Crude OR (99% Cl)**	**Adjusted OR^a^ (99% CI)**	**Adjusted OR^b^ (99% CI)**
**Variable**		**Overall (*N* = 240)**	**High practise (*N* = 28)**	**Low practise *N* = 212)**				
Gender	Male	1 (0.42)	0 (0)	1 (0.47)	1	1	Omitted	NA
	Female	239 (99.6)	28 (100)	211 (99.53)		Omitted	NA	NA
Age	Mean (SD)	35.5 (11.8)	35.11 (10.66)	35.53 (11.92)	0.85	1.00 (0.95–1.04)	1.00 (0.96–1.05)	1.01 (0.96–1.05)
	Median (IQR)	32 (26–44); Range: 18–71	32.5 (26.5–44.5); Range: 20–55	32 (26–44); Range: 18–71	0.98	NA		
	≤ 30	110 (46.03)	12 (42.86)	98 (46.45)	0.82	1	1	1
	31–50	96 (40.17)	13 (46.43)	83 (39.34)		0.82 (0.14–4.69)	1.01 (0.16–6.24)	1.06 (0.17–6.61)
	>50	33 (13.8)	3 (10.71)	30 (14.22)		1.28 (0.43–3.85)	1.66 (0.51–5.43)	1.68 (0.51–5.50)
Ethnicity	Chinese	125 (52.1)	12 (42.86)	113 (53.3)	0.06	1	1	1
	Malay	56 (23.3)	8 (28.57)	48 (22.64)		1.57 (0.45–5.51)	1.79 (0.48–6.75)	1.66 (0.43–6.38)
	Indian	28 (11.7)	7 (25)	21 (9.91)		3.14 (0.80–12.35)ramyaI!!	4.43 (1.03–19.04)^*^	4.65 (1.07–20.26)^*^
	Others	31 (12.9)	1 (3.57)	30 (14.15)		0.31 (0.02–4.83)	0.28 (0.01–5.68)	0.26 (0.01–5.43)
Highest educational qualification	Pre-Primary/Primary/Secondary	49 (20.42)	8 (28.57)	41 (19.34)	0.52	1	1	1
	Post-secondary (Non-tertiary)	20 (8.3)	3 (10.71)	17 (8.02)		0.90 (0.14–6.02)	0.90 (0.13–6.47)	0.95 (0.13–6.94)
	Diploma courses	99 (41.3)	11 (39.29)	88 (41.51)		0.64 (0.18–2.33)	0.59 (0.15–2.27)	0.59 (0.15–2.31)
	University & Above	72 (30)	6 (21.43)	66 (31.13)		0.47 (0.11–2.05)	0.46 (0.10–2.12)	0.47 (0.10–2.19)
Number of years worked in CCC	Mean (SD)	6.48 (6.79) ()	7.25 (8.97) ()	6.38 (6.46) ()	0.62	1.02 (0.95–1.09)	1.01 (0.94–1.08)	1.01 (0.94–1.09)
	Median (IQR)	4 (2–9); Range: 0–40	4.5 (1.5–9.5); Range (1–40)	4 (2–9); Range: 0–38	0.97	NA		
	< 1	15 (6.3)	0 (0)	5 (7.18)	0.40	1	1	1
	1–5	121 (51.05)	16 (57.14)	105 (50.24)		1.60 x 10^6^ (0–NE)	1.75 x 10^6^ (0–NE)	2.79 x 10^6^ (0–NE)
	>5	101 (42.62)	42 (42.86)	89 (42.58)		1.41 x 10^6^ (0–NE)	1.33 x 10^6^ (0–NE)	2.14 x 10^6^ (0–NE)
Part-time / Full-time status	Full–time	228 (95)	26 (92.86)	202 (95.28)	0.42	1	1	1
	Part–time	12 (5)	2 (7.14)	10 (4.72)		1.55 (0.20–12.27)	1.28 (0.16–10.59)	1.25 (0.15–10.37)
Type of childcare	Public	70 (29.2)	11 (39.29)	59 (27.83)	0.27	1	1	1
	Non–Public (Private/Autonomous)	170 (70.83)	17 (60.71)	153 (72.17)		0.60 (0.20–1.74)	0.49 (0.16–1.51)	0.49 (0.16–1.52)
Staff/Children ratio	Mean (SD)	11.34 (6.03) ()	10.93 (6.11) ()	11.45 (6.03) ()	0.68	0.99 (0.90–1.08)	0.96 (0.87–1.06)	0.96 (0.87–1.06)
	Median (IQR)	10 (7–15); Range: 0–28	8.5 (7.5–15); Range: 0–25	10 (7–15); Range: 0–28	0.73	NA		
	1–5	52 (21.8)	5 (17.86)	47 (22.27)	0.85	1	1	1
	6–15	139 (58.16)	18 (64.29)	121 (57.35)		1.40 (0.35–5.53)	1.05 (0.25–4.36)	1.05 (0.25–4.36)
	16–28	48 (20.08)	5 (17.86)	43 (20.38)		1.09 (0.20–6.09)	0.75 (0.12–4.70)	0.75 (0.12–4.72)
Have children	Yes	116 (48.3)	16 (57.14)	100 (47.17)	0.42	1	1	1
	No	124 (51.7)	12 (42.86)	112 (52.83)		0.67 (0.24–1.91)	0.46 (0.14–1.47)	0.44 (0.14–1.42)
Number of children (if any)	1	49 (42.2)	5 (31.25)	44 (44)	0.65	1	1	1
	2	37 (31.9)	6 (37.5)	31 (31)		1.70 (0.32–9.07)	1.49 (0.25–8.73)	1.48 (0.25–8.94)
	≥3	30 (25.86)	5 (31.25)	25 (25)		1.76 (0.31–10.15)	1.88 (0.29–11.97)	1.84 (0.29–11.82)
Send children to CCC	Yes	59 (51.8)	11 (68.75)	48 (48.98)	0.18	1	1	1
	No	55 (48.3)	5 (31.25)	50 (51.02)		0.44 (0.10–1.92)	0.56 (0.12–2.67)	0.55 (0.11–2.64)

OR, Odds ratio;

* p < 0.01; ramyaI!!0.01 ≤ p < 0.05; NA, Not Applicable; NE, Not Estimable; CCC, childcare centre p, Fisher's exact test p-value (categorical variable) or t-test p-value (continuous variable).

a adjusted for Q6 Score, Q20 Score.

b adjusted for Q6 Score, Q20 Score, HFMD-related centre closure experience (Yes as reference group).

**Figure 1:**
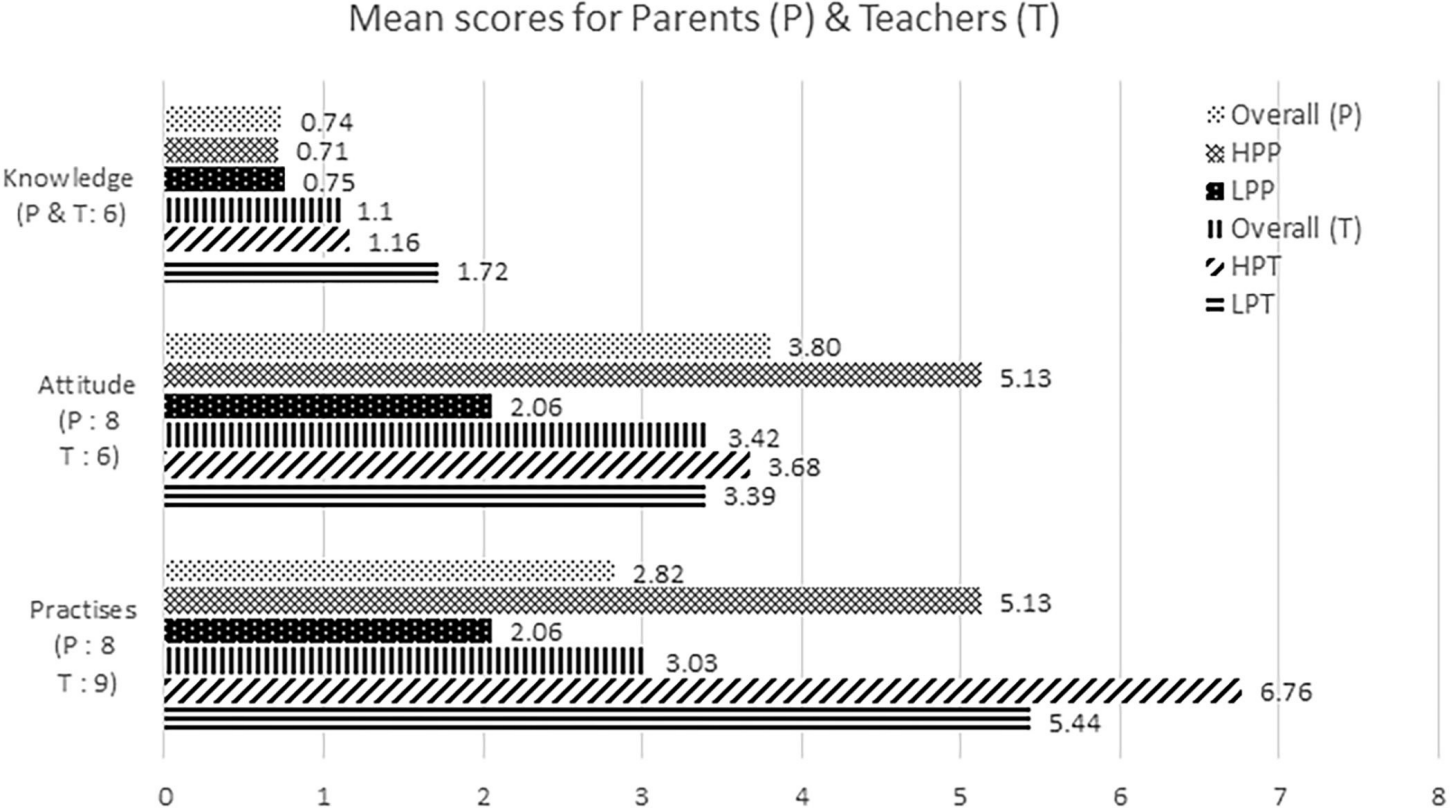
KAP scores of parents and teachers. The highest scores that can be scored by the parents (P) and teachers (T) for each domain are denoted in brackets, respectively. HPP, High practise parents; LPP, Low practise parents; HPT, High practise teachers; LPT, Low practise teachers. (Between high and low practise groups).

The key findings of this study are summarised in Table 3, and the mean scores by each population for each domain are shown in Figure 1.

**Table 3:** Key study findings on HFMD-PS KAP and facilitators.

**Domain**	**Parents**	**Teachers**
Knowledge	1. Overall good knowledge – Overall mean score a. Parents: 0.74 ± 1.76 b. Teachers: 1.10 ± 1.76 2. Similarly low but positive knowledge scores across high practise and low practise groups a. HPP 0.71 ± 1.75, LPP 0.75 ± 1.77, *p* = 0.86 b. HPT 0.65 ± 2.01, LPT 1.16 ± 1.72, *p* = 0.21 3. In all parents and teachers, regardless of practise level a. Poor knowledge on HFMD transmission modes and infectious period and b. Low awareness of regular soap and household bleach's efficacy as a disinfectant	
Attitude	1. Overall receptive attitudes—overall mean score 3.80 ± 1.63 2. Positive but low levels of attitude scores in both groups, though HPP had significantly higher attitude scores (HPP 5.13 ± 0.7, LPP 2.06 ± 1.88, p < 0.01) 3. HPP were significantly more receptive towards a. Regular cleaning or disinfection of toys and high-frequency contact surfaces and b. Washing of hands (self or child's) in accordance with the seven-step handwashing technique	1. Overall receptive attitudes—overall mean score 3.42 ± 1.67 2. In both high and low practise groups, a. Similarly low but positive attitude scores across groups (HPT 3.68 ± 1.41, LPT 3.39 ± 1.71, p=0.33) b. Similar levels of receptiveness towards i. Regular cleaning and disinfection of toys and high-contact surfaces ii. School closures due to HFMD outbreaks in centres iii. Acquiring more HFMD-related knowledge
Practises	1. Overall positive practise levels—overall mean score 2.82 ± 2.13 2. HPP has significantly higher scores than LPP (HPP 5.13 ± 0.70, LPP 2.06 ± 1.88, p < 0.01) 3. In both groups, there is a low preference for household bleach as disinfectant 4. Between groups, there are significantly different practise levels in a. Frequency of cleaning and cleaning agents used to clean toys and homes b. Compliance to proper handwashing steps c. Response to keeping an infected child at home	1. Overall positive practise levels—overall mean score 3.03 ± 1.21 2. HPT has significantly higher scores than LPT for a. Overall practise scores—HPT 6.76 ± 0.15, LPT 5.44 ± 1.11, (p < 0.01) b. Compliance to proper handwashing steps (whether self or child's) 3. No significant difference in scores and responses chosen for all other practises assessed
Facilitators for high levels of HFMD-PS	1. Cleaning agent used to clean toys a. Q11 adjusted OR with HFMD experience 56.7, 99% CI 7.06-455.61) b. With warm water and detergent—Q11 adjusted OR 3.61, 99% CI 1.81-7.21 2. Daily toy cleaning, before and after playtime a. Q9 adjusted OR 4.72, 99% CI 1.28-17.4) 3. Awareness of regular liquid soap's efficacy as a disinfectant a. adjusted OR with HFMD experience 2.18, 99% CI 1.09-4.33 4. Sending child to a non-public childcare centre a. adjusted OR 1.91, 99% CI 0.94-3.86) 5. Never experiencing a HFMD episode in their children a. adjusted OR 1.81, 99% CI 0.94-3.53)	1. Indian ethnicity, regardless of centre's HFMD experience a. adjusted OR 4.43, 99% CI 1.03-19.04 b. adjusted OR with HFMD experience 4.65, 99% CI 1.07-20.26

### Parents: Knowledge, attitudes, and practise of HFMD and its prevention strategies

Parents generally had not a high level of knowledge of HFMD (overall mean 0.74 ± 1.76, Table 4) but a positive attitude towards implementing good practises to prevent HFMD (overall mean 3.80 ± 1.63, Supplementary Table 5). However, LPP had significantly lower attitude scores than HPP, although both groups had positive but low levels of attitude scores (HPP 5.13 ± 0.7, LPP 2.06 ± 1.88, *p* < 0.01, Supplementary Table 5). Despite positive, overall HFMD knowledge scores were also similarly low between the groups (HPP 0.71 ± 1.75, LPP 0.75 ± 1.77, *p* = 0.86, Table 4). Similarly, parents generally practised HFMD-PS as practise domain scores were positive in both groups (overall mean 2.82 ± 2.13, Supplementary Table 6). However, the compliance with these strategies was notably higher amongst HPP, relative to LPP (HPP 5.13 ± 0.70, LPP 2.06 ± 1.88, *p* < 0.01, Supplementary Table 6).

**Table 4:** Knowledge of parents and teachers towards HFMD.

**Knowledge**		**Number of respondents (%)**			***p***	**Crude OR (99% Cl)**	**Adjusted OR^a^ (99% CI)**	**Adjusted OR^b^ (99% CI)**
**Question**	**Response options**	**Overall**	**High practise**	**Low practise**				
**Parents**		***N* = 404**	***N* = 99**	***N* = 305**				
Overall Score	Mean (SD)	0.74 (1.76)	0.71 (1.75)	0.75 (1.77)	0.86	0.99 (0.83–1.17)	1.09 (0.90–1.33)	1.09 (0.90–1.33)
	Median (IQR)	0.8 (−0.1–2.2); Range: −5–5	0.6 (−1–2.15); Range: −3.6–5	0.8 (0.15–2.2); Range: −5–5	0.75	NA		
1	Non–Viral	114 (28.22)	28 (28.28)	86 (28.2)	1	1	1	1
	Viral	290 (71.78)	71 (71.72)	219 (71.8)		1.00 (0.51–1.93)	1.31 (0.63–2.71)	1.30 (0.63–2.70)
	Q1 Score: Mean (SD)	0.44 (0.90)	0.44 (0.90)	0.44 (0.90)	0.99	1.00 (0.72–1.39)	1.14 (0.79–1.65)	1.14 (0.79–1.64)
2	Saliva	339 (83.91)	79 (79.8)	260 (85.25)	0.21	0.68 (0.32–1.47)	0.98 (0.42–2.30)	0.97 (0.41–2.27)
	Stool	186 (46.04)	39 (39.39)	147 (48.2)	0.13	0.70 (0.38–1.28)	0.83 (0.43–1.63)	0.87 (0.44–1.71)
	Fluid from an infected person's blisters	318 (78.71)	76 (76.77)	242 (79.34)	0.58	0.86 (0.42–1.76)	1.10 (0.5–2.42)	1.14 (0.51–2.51)
	Respiratory droplets	301 (74.5)	76 (76.77)	225 (73.77)	0.60	1.17 (0.58–2.36)	1.48 (0.69–3.19)	1.58 (0.73–3.42)
	Touching surfaces previously touched by infected case	293 (72.52)	78 (78.79)	215 (70.49)	0.12	1.55 (0.76–3.17)	1.25 (0.58–2.72)	1.29 (0.59–2.82)
	I don't know	3 (0.74)	0 (0)	3 (0.98)	1	Omitted	Omitted	Omitted
	Q2 Score: Mean (SD)	−0.54 (0.77)	−0.65 (0.70)	−0.51 (0.78)	0.11	0.78 (0.52–1.19)	0.89 (0.57–1.40)	0.88 (0.55–1.39)
3	Alcohol–based sanitiser	319 (78.96)	83 (83.84)	236 (77.38)	0.20	1.52 (0.69–3.33)	1.60 (0.69–3.72)	1.60 (0.69–3.73)
	Regular Liquid Soap	205 (50.74)	56 (59.6)	146 (47.87)ramyaI!!	0.05	1.61 (0.88–2.94)ramyaI!!	2.23 (1.12–4.42)^*^	2.18 (1.09–4.33)^*^
	Antibacterial liquid soap	309 (76.49)	81 (81.82)	228 (74.75)	0.17	1.52 (0.72–3.22)	1.38 (0.61–3.10)	1.39 (0.61–3.15)
	Hospital grade soap	250 (61.88)	64 (64.65)	186 (60.98)	0.55	1.17 (0.63–2.18)	1.28 (0.65–2.55)	1.24 (0.62–2.46)
	Household bleach	177 (43.81)	48 (48.48)	129 (42.3)	0.30	1.28 (0.71–2.33)	1.84 (0.93–3.64)ramyaI!!	1.81 (0.91–3.58)ramyaI!!
	I don't know	27 (6.68)	3 (3.03)	24 (7.87)	0.11	0.37 (0.07–1.82)	0.42 (0.08–2.29)	0.46 (0.08–2.57)
	Q3 Score: Mean (SD)	0.55 (0.49)	0.64 (0.39)	0.52 (0.51)ramyaI!!	0.02	1.82 (0.87–3.81)ramyaI!!	2.27 (0.94–5.50)ramyaI!!	2.19 (0.90–5.32)ramyaI!!
4	Fever	373 (92.33)	91 (91.92)	282 (92.46)	0.83	0.93 (0.31–2.79)	1.04 (0.32–3.43)	1.08 (0.32–3.60)
	Blisters on the hands and feet	398 (98.51)	97 (97.98)	301 (98.69)	0.64	0.64 (0.07–6.12)	0.64 (0.06–6.66)	0.69 (0.06–7.93)
	Mouth ulcers	400 (99.01)	97 (97.98)	303 (99.34)	0.25	0.32 (0.02–4.28)	0.23 (0.02–3.19)	0.27 (0.02–3.80)
	Poor appetite	59 (14.6)	16 (16.16)	43 (14.1)	0.63	1.17 (0.52–2.67)	1.56 (0.63–3.93)	1.50 (0.59–3.81)
	Vomiting	39 (9.65)	12 (12.12)	27 (8.85)	0.33	1.42 (0.55–3.66)	1.93 (0.67–5.62)	1.75 (0.60–5.17)
	Lethargy	67 (16.58)	21 (21.21)	46 (15.08)	0.16	1.52 (0.71–3.22)	1.75 (0.76–4.02)	1.78 (0.77–4.11)
	I don't know	77 (19.06)	20 (20.2)	57 (18.69)	0.77	1.10 (0.52–2.33)	1.44 (0.63–3.28)	1.43 (0.62–3.29)
	Q4 Score: Mean (SD)	0.50 (0.03)	0.51 (0.20)	0.50 (0.17)	0.51	1.59 (0.31–8.31)	2.90 (0.46–18.34)	2.76 (0.43–17.68)
5	Not false	50 (12.38)	14 (14.14)	36 (11.8)	0.60	1	1	1
	False	354 (87.62)	85 (85.86)	269 (88.2)		0.81 (0.34–1.94)	0.87 (0.32–2.33)	1.07 (0.38–2.99)
	Q5 Score: Mean (SD)	0.75 (0.66)	0.72 (0.70)	0.76 (0.65)	0.56	0.90 (0.58–1.39)	0.93 (0.57–1.53)	1.04 (0.62–1.73)
6	Not True	294 (72.77)	71 (71.72)	223 (73.11)	0.80	1	1	1
	True	110 (27.23)	28 (28.28)	82 (26.89)		1.07 (0.55–2.08)	1.25 (0.60–2.61)	1.21 (0.58–2.53)
	Q6 Score: Mean (SD)	−0.46 (0.89)	−0.43 (0.91)	−0.46 (0.89)	0.79	1.04 (0.74–1.44)	1.12 (0.78–1.62)	1.10 (0.76–1.59)
**Teachers**		***N*** **=** **240**	***N*** **=** **28**	***N*** **=** **212**				
Overall Score	Mean (SD)	1.10 (1.76) ()	0.65 (2.01) ()	1.16 (1.72) ()	0.21	0.85 (0.64–1.13)	0.93 (0.68–1.28)	0.93 (0.67–1.28)
	Median (IQR)	1.27 (0–2); Range: −6–5.47	1.05 (−0.64–1.83); Range: −4.5–3.75	1.27 (0–2); Range: −6–5.47	0.25	NA		
1	Non–Viral	67 (27.92)	9 (32.14)	58 (27.36)	0.66	1	1	1
	Viral	173 (72.08)	19 (67.86)	154 (72.64)		0.80 (0.26–2.43)	0.83 (0.26–2.65)	0.84 (0.26–2.66)
	Q1 Score: Mean (SD)	0.44 (0.90)	0.36 (0.95)	0.45 (0.89)	0.62	0.89 (0.51–1.56)	0.91 (0.51–1.63)	0.91 (0.51–1.63)
2	Saliva	195 (81.59)	22 (81.48)	173 (81.6)	1	0.99 (0.26–3.85)	1.13 (0.28–4.63)	1.11 (0.27–4.57)
	Stool	111 (46.44)	13 (48.15)	98 (46.23)	1	1.08 (0.38–3.10)	1.18 (0.39–3.59)	1.17 (0.38–3.58)
	Fluid from an infected person's blisters	201 (84.1)	23 (85.19)	178 (83.96)	1	1.10 (0.25–4.81)	1.82 (0.37–9.05)	1.76 (0.35–8.84)
	Respiratory droplets	173 (72.38)	18 (66.67)	155 (73.11)	0.50	0.74 (0.24–2.26)	0.76 (0.23–2.44)	0.73 (0.22–2.37)
	Touching surfaces previously touched by infected case	183 (76.57)	22 (81.48)	161 (75.94)	0.64	1.39 (0.36–5.33)	1.77 (0.41–7.54)	1.79 (0.42–7.67)
	I don't know	1 (0.42)	0 (0)	1 (0.47)	1	Omitted	Omitted	Omitted
	Q2 Score: Mean (SD)	−0.62 (0.71)	−0.69 (0.68)	−0.61 (0.72)	0.58	0.85 (0.39–1.87)	0.72 (0.30–1.72)	0.72 (0.30–1.71)
3	Alcohol–based sanitiser	174 (72.5)	20 (71.43)	154 (72.64)	1	0.94 (0.30–2.97)	1.03 (0.31–3.40)	0.99 (0.3–3.32)
	Regular Liquid Soap	96 (40)	13 (46.43)	83 (39.15)	0.54	1.35 (0.48–3.82)	1.43 (0.48–4.26)	1.40 (0.47–4.19)
	Antibacterial liquid soap	181 (75.42)	17 (60.71)	164 (77.36)	0.06	0.45 (0.15–1.34)	0.58 (0.18–1.83)	0.56 (0.17–1.78)
	Hospital grade soap	139 (57.92)	12 (42.86)	127 (59.91)	0.10	0.50 (0.18–1.43)	0.55 (0.18–1.64)	0.54 (0.18–1.63)
	Household bleach	88 (36.67)	11 (39.29)	77 (36.32)	0.84	1.13 (0.39–3.28)	1.45 (0.47–4.45)	1.47 (0.48–4.52)
	I don't know	14 (5.83)	3 (10.71)	11 (5.19)	0.22	2.19 (0.38–12.8)	1.90 (0.31–11.58)	2.00 (0.32–12.27)
	Q3 Score: Mean (SD)	0.51 (0.46)	0.41 (0.59)	0.52 (0.44)	0.38	0.65 (0.24–1.76)	0.76 (0.27–2.19)	0.74 (0.26–2.14)
4	Fever	209 (87.08)	21 (75)	188 (88.68)	0.07	0.38 (0.11–1.34)ramyaI!!	0.38 (0.10–1.45)	0.38 (0.10–1.45)
	Blisters on the hands and feet	238 (99.17)	28 (100)	210 (99.06)	1	Omitted	Omitted	Omitted
	Mouth ulcers	230 (95.83)	25 (89.29)	205 (96.7)	0.10	0.28 (0.04–1.83)	0.41 (0.06–3.11)	0.43 (0.06–3.26)
	Poor appetite	168 (70)	15 (53.57)	153 (72.17)	0.05	0.44 (0.16–1.28)	0.49 (0.16–1.46)	0.48 (0.16–1.45)
	Vomiting	70 (29.17)	6 (21.43)	64 (30.19)	0.39	0.63 (0.18–2.20)	0.63 (0.17–2.29)	0.57 (0.15–2.16)
	Lethargy	101 (42.08)	8 (28.57)	93 (43.87)	0.16	0.51 (0.16–1.59)	0.45 (0.13–0.15)	0.45 (0.13–1.50)
	I don't know	1 (0.42)	0 (0)	1 (0.47)	1	Omitted	Omitted	Omitted
	Q4 Score: Mean (SD)	0.7 (0.25)	0.61 (0.27)	0.71 (0.25)	0.07	0.26 (0.04–1.67)	0.29 (0.05–1.89)	0.28 (0.04–1.82)
5	Not false	33 (13.75)	2 (7.14)	31 (14.62)	0.39	1	1	1
	False	207 (86.25)	26 (92.86)	181 (85.38)		2.23 (0.32–15.73)	2.36 (0.32–17.54)	2.34 (0.32–17.45)
	Q5 Score: Mean (SD)	0.73 (0.69)	0.86 (0.52)	0.71 (0.71)	0.18	1.49 (0.56–3.97)	1.54 (0.56–4.19)	1.53 (0.56–4.18)
6	Not True	199 (82.92)	27 (96.43)	172 (81.13)	0.06	1	1	1
	True	41 (17.08)	1 (3.57)	40 (18.87)		0.16 (0.01–2.28)	Omitted	Omitted
	Q6 Score: Mean (SD)	−0.66 (0.75)	−0.93 (0.38)	−0.62 (0.78)	< 0.01	0.40 (0.11–1.51)	NA	NA

OR, Odds ratio;

* p < 0.01; ramyaI!!0.01 p < 0.05; NA, Not Applicable; p, Fisher's exact test p-value (categorical variable) or t-test p-value (continuous variable).

a Parents: adjusted for ethnicity, Q16 Score, Q17 Score; Teachers: adjusted for Q6 Score, Q20 Score.

b Parents: adjusted for ethnicity, Q16 Score, Q17 Score, HFMD Status (Yes as reference group); Teachers: adjusted for Q6 Score, Q20 Score, HFMD-related centre closure experience (Yes as reference group).

#### Knowledge

HPP had a non-significantly higher score in their knowledge for various disinfectant's effectiveness in removing HFMD viruses than the LPP (Q3 mean score, HPP 0.64 ± 0.39, LPP 0.52 ± 0.51, *p* = 0.015, Table 4). The difference in mean scores could be driven by an additional 11.7% HPP correctly identifying regular liquid soap as an effective disinfectant, compared with LPP (HPP 59.6%, LPP 47.9%, Fisher's *p* = 0.049, Table 4). Nonetheless, poor knowledge of HFMD transmission modes and infectious period was observed in both HPP and LPP. Parents generally failed to identify stool as a potential source of infection (overall 46.0%, HPP 39.4%, LPP 48.2%, Fisher's *p* = 0.13, Table 4), but instead only identified touching surfaces previously touched by someone infected as a potential source of infection (overall 75.5%, HPP 78.8%, LPP 70.5%, Fisher's *p* = 0.12, Table 4). In addition, ~73% parents, regardless of HPP or LPP, failed to identify that HFMD's infectious period can go beyond a child's symptomatic period (overall 72.8%, HPP 71.7%, LPP 73.1%, Table 4).

#### Attitude

In general, LPP was more receptive towards implementing HFMD-PS, except towards the 10-day school closure in response to a HFMD outbreak at a centre. Compared with HPP, a non-significant but higher percentage of LPP generally felt that a 10-day school closure was ‘too long' (HPP 27.3%, LPP 35.7%, Fisher's *p* = 0.14), despite the epidemiologically justified 10-day period to break an ongoing transmission. Similarly, a significantly higher proportion of LPP felt that the school closures were too much of a burden (HPP 12.1%, LPP 31.2%, Fisher's *p* < 0.0001), even though at least 50% reported being able to cope with the inconvenience by making necessary arrangements (HPP 45.5%, LPP 57.4%, Fisher's *p* = 0.048). Nonetheless, the collective attitudes regarding school closure measures to control HFMD spread did not differ significantly across groups (Q19a mean score *p* = 0.09, Q19b mean score *p* = 0.15, Supplementary Table 5). Parents in both groups were also generally receptive towards vaccinating their children against HFMD, if available (overall 65.4%, HPP 68.7%, LPP 64.3%, Supplementary Table 5). Amongst parents who neither readily agreed nor disagreed to vaccinating their children against HFMD (32.2%, Supplementary Table 5), vaccine safety (26.4%, Supplementary Table 5), and efficacy (24.5%) were the most common concerns. At least 50% of the parent respondents were willing to learn more about HFMD (58.2%, Supplementary Table 5), with a preference to do so through brochures (61.3%, Supplementary Table 5) and social media (67.7%).

Both HPP and LPP expressed positive attitudes towards regular cleaning or disinfecting their children's toys and high-frequency contact surfaces and washing their own or children's hands in compliance with the seven-step handwashing technique. However, these strategies were more positively received by HPP compared with LPP (Q15–18 mean scores *p* < 0.01, Supplementary Table 5). Compared with their counterparts, positive and non-positive attitudes towards these strategies were consistently expressed by higher proportions of the HPP and LPP groups, respectively. The difference in proportion between groups was always significant or almost reached statistical significance, when related to regular cleaning or disinfection of toys or high-contact surfaces (Fisher's *p*-value = 0.03 to < 0.01, Supplementary Table 5). However, it may not always be significant for specific attitudes towards compliance with proper handwashing steps. Notably, a higher proportion of parents in the HPP group believe that following the handwashing steps to properly wash hands is important (own hands HPP 79.8%, LPP 64.9%, Fisher's *p* < 0.01; children's hands HPP 81.8%, LPP 65.3%, Fisher's *p* < 0.01, Supplementary Table 5) and not tedious (own hands HPP 89.1%, LPP 75.1%, Fisher's *p* = 0.002; children's hands HPP 88.9%, LPP 69.5%, Fisher's *p* < 0.01, Supplementary Table 5). A higher proportion of parents in the HPP group also believe in the protective effect of following proper handwashing steps and in reducing infectious disease infection risk amongst their children (HPP 73.7%, LPP 55.7%, Fisher's *p* < 0.01).

#### Practises

Parents generally had positive practises against HFMD infection, but there was a low preference for using bleach as a cleaning agent (Q13 overall mean score −0.10 ± 0.79, Supplementary Table 6). Almost half of LPP reportedly never used bleach as a cleaning agent (LPP 53.1%, LPP Q13 mean score −0.31 ± 0.76, Supplementary Table 6), although 42.3% of them correctly identified bleach as an effective disinfectant (Table 4). In contrast, using bleach as a cleaning agent at least once a week was the most common response amongst HPP (45.5%, HPP Q13 mean score 0.52 ± 0.46, Supplementary Table 6). Other significantly different practises include the following: (1) washing their own or their children's hands according to the proper handwashing steps, (2) frequency of cleaning and agents used to clean their children's toys and house, and (3) their response towards keeping their child at home upon receiving their child's medical certificate for HFMD infection. The consistency in practising proper handwashing steps between HPP and LPP was also evidently different. A majority of HPP always followed the steps (58.6%, Supplementary Table 6), while a majority of LPP did not always follow the steps (76.7%, Supplementary Table 6).

### Teachers: Knowledge, attitudes, and practise of HFMD and its prevention strategies

Teachers had not a high level of knowledge of HFMD (overall mean 1.10 ± 1.76, Table 4) but a positive attitude towards implementing good practises to prevent HFMD (overall mean 3.42 ± 1.67, Supplementary Table 5). Similar to the parent sub-population, LPT and HPT had positive but low overall attitude scores (HPT 3.68 ± 1.41, LPT 3.39 ± 1.71, *p* = 0.33, Supplementary Table 5) and low levels of HFMD-related knowledge (HPT 0.65 ± 2.01, LPT 1.16 ± 1.72, *p* = 0.21, Table 4). Teachers also practised HFMD-PS sufficiently, with positive overall practise scores in both groups (HPT 6.76 ± 0.15, LPT 5.44 ± 1.11, *p* < 0.00001, Supplementary Table 6). Interestingly, LPTs were slightly more knowledgeable about HFMD, although their actual level of practising HFMD-PS was notably lower than HPTs.

*Knowledge* Good overall knowledge of HFMD was observed amongst teachers, with positive scores reported across all questions except those on the modes of HFMD's transmission (Q2 mean score −0.62 ± 0.71, Table 4) and infectious period (Q6 mean score −0.66 ± 0.75, Table 4). Poor knowledge in these areas was also observed in parents and even amongst HPTs (Q2 mean score −0.69 ± 0.68, Q6 mean score −0.93 ± 0.38, Table 4). However, overall Q6 score was significantly higher for HPT than for LPT (LPT −0.62 ± 0.78, *p* = 0.0011, Table 4). The proportion of HPT (*n* = 22, 12%) amongst the 183 teachers who incorrectly identified ‘touching surfaces previously in contact with an infected case' as a mode of transmission was much lower than that of LPT (88%). Likewise, HPTs were the minority of the 199 teachers who identified the non-positive options for HFMD's infectious period (‘False' or ‘I don't know'). Amongst all teachers, the efficacy of household bleach and regular liquid soap as a disinfectant was also not widely known compared with that of other cleaning agents (bleach 36.7%, regular liquid soap 40%, Table 4). The low awareness was consistent with that observed amongst parents and even amongst HPT (bleach 39.3%, regular liquid soap 46.4%, Table 4). The difference in awareness of household bleach's efficacy between HPT and LPT was slight (HPT 39.3%, LPT 36.3%, Table 4) but more notable for regular liquid soap (HPT 46.4%, LPT 39.2%, Table 4). However, the differences in awareness were not statistically significant (Fisher's p: bleach = 0.834, regular liquid soap = 0.54).

#### Attitude

Teachers had supportive attitudes towards HFMD prevention practises, with positive mean scores for all questions in HPT and LPT. Question-specific scores were consistently higher in HPT compared with LPT, except for questions relating to their attitudes towards regular cleaning and disinfecting toys and high-contact surfaces (Q13 and Q14, Supplementary Table 5). However, the difference in scores was all non-significant (Fisher's *p*-value = 0.08 (Q10 score) to 0.91 (Q27 score), Supplementary Table 5). This indicates similar attitudes between teachers in both groups in terms of (1) regular cleaning and disinfecting toys and high-contact surfaces, (2) school closures in response to HFMD outbreaks in their centres, and (3) learning more about HFMD. It was interesting to note that mixed attitudes towards regular cleaning of toys and high-contact surfaces were expressed by more LPT compared with HPT. However, the difference in prevalence was marginal (Fisher's *p*-value = 0.041 (‘important' for Q14) to 1.00 (‘tedious', ‘excessive', and others: for Q13, ‘excessive' for Q14), Supplementary Table 5).

#### Practise

Although HPT and LPT expressed similar attitudes towards complying to proper handwashing steps, HPT scored significantly higher than LPT for the questions assessing this practise (HPT mean score (Q7 and Q9) 1.0 ± 0, LPT mean score (Q7) 0.75 ±0.3, LPT mean score (Q9) 0.82 ± 0.29, *p* < 0.01, Supplementary Table 6). Nonetheless, a larger proportion of LPT reported ‘always' practising handwashing according to proper handwashing steps when washing their own hands (60.4%) or when assisting the children with handwashing (70.8%) compared with HPT (50% for own hands, 67.9% for assisting children, Supplementary Table 6). However, a higher frequency of cleaning toys, i.e., cleaning at least once a day as recommended by guidelines, was more commonly reported by HPT (67.9%) than LPT (61.8%). Ineffective pathogen removal from toys was also more prevalent amongst LPT than HPT (dry wipe, HPT 3.6%, LPT 13.2%; wipe with wet cloth, HPT 10.7%, LPT 18.9%, Supplementary Table 6). While most parents kept their HFMD-infected child home for the full duration indicated by the medical certificate, a small proportion of teachers reported encountering parents failing to do so (34.2%, Supplementary Table 6). A relatively higher proportion of HPT reported encountering such situations (42.9%) compared with LPT (33.0%). However, all HPT encountering such situation refused the child entry. These HPT instead opted to educate the parent and deny the child entry (83.3%) unless a medical endorsement (83.3%) or principal approval (16.7%) to allow the child entry was obtained. LPT in such situations more often asked for medical endorsement (88.6%), rather than educate the parent and deny the child entry (68.6%). LPT also sought principal approval more often (30%) and one LPT reportedly allowed the child to enter (0.50%). However, it was likely that the child's entry was granted only after obtaining medical endorsement or principal approval, as the same teacher also reported practising all other measures provided in this sub-question. In addition, it is interesting to note that LPT scored slightly higher than HPT on practises when a child starts showing symptoms during the day (mean score, overall 0.46 ± 0.13, HPT 0.38 ± 0.21, LPT 0.47 ± 0.11, *p* = 0.04, Supplementary Table 6). Nonetheless, differences in scores and options chosen for all practise questions were not significantly different between HPT and LPT. The only exception was for scores on the frequency of compliance with proper handwashing steps (Q7 and Q9), even though differences in frequency between LPT and HPT were not statistically significant (Fisher's *p*-value = 0.127 to 0.826, Supplementary Table 6).

### Teachers: Experience of HFMD episodes or outbreaks in centres

Amongst the 240 respondents, 78 teachers (overall 32.5%, HPT 32.14%, LPT 32.55%) responded ever facing difficulties in getting parents to comply with the school's decision (Q19). Reasons cited by these parents were commonly work related and/or a lack of alternative care arrangements for the child, especially at short notice. The remaining parents refused to comply as they were in denial of their child's condition, and believe their child is well-or just mildly unwell. Overall, only 6.25% teachers (*n* = 15) reported experiencing centre closures due to HFMD outbreak. Difficulties faced by these teachers during the closure were mainly: (1) attending to the parents' concerns and complaints about the school closure, (2) the additional workload from required sanitisation of the classroom and all materials including toys, and (3) potential problems in completing the curriculum due to delayed lessons. It was interesting to note that 78 teachers also anticipated potential difficulties with a centre closure, even though they have yet to experience it first-hand. This group of teachers also highlighted the following: (1) the need to conduct lessons online, (2) additional burden from daily checking in with each child's parent to track the child's health condition and update them on the outbreak progression, (3) assuring the parents of the centre's safety after the closure is over, and even (4) a loss in rapport between the child and teacher after the child is allowed to come back to school as potential difficulties. Other challenges faced by teachers when dealing with HFMD cases in the school include the following: (1) delayed medical diagnosis, (2) confusing symptoms (such as eczema that look like blisters on hands or a concurrent gastritis outbreak in the school), (3) verbal abuse by parents when they believe that their child is alright or that their child was infected in the school, and (4) worry about spreading the disease to their loved ones at home.

To support infection control policies, teachers suggested educating parents and reinforcing rules to promote parent cooperation in preventing HFMD episodes and outbreaks. This includes the following: (1) increasing the mandated amount of parent care leave (commonly complaints about lacking alternative care arrangement and insufficient leaves to take care of their children at home), (2) educating parents on HFMD-related knowledge (e.g., common symptoms and infection control policies in childcare centres) and having stricter rules to ensure parents observe the regulations, (4) enforcing school policies on daily health cheques at drop-off, and (5) including guidelines to keep the siblings of the infected child home even if they are asymptomatic currently. Teachers also mentioned the need to educate parents on the importance of good hand hygiene practises with their children at home and improve the school's cleanliness by engaging professional disinfection services to clean the entire centre during centre closures.

### Facilitators for practising high levels of HFMD-PS

Potential facilitators for good practise of HFMD-PS in each sub-population were identified after adjusting for the respective confounders. Confounders in the parent sub-population include ethnicity, and question scores relating to attitudes towards getting children to comply with proper handwashing steps (Q16) and regular disinfection of their children's toys (Q17). Confounders in the teacher sub-population include the question scores for knowledge of HFMD's infectious period (Q6) and actions taken when a child turns symptomatic during the school session (Q20).

#### Parent

Facilitators for practising a high level of HFMD-PS in parents include sending their children to non-public childcare centres (adjusted OR 1.91, 99% CI 0.94–3.86, Table 1) and never experiencing a HFMD episode in their children (adjusted OR 1.81, 99% CI 0.94-3.53, Table 1). Knowledge of effective disinfectants (Q3) also increases the likelihood of high adoption levels of HFMD-PS by 2.27 times, for every unit increase in knowledge (Q3 adjusted OR 2.27, 99% CI 1.12–4.42, Table 4). The strong association between Q3 score and higher practise levels of HFMD-PS could be driven by the correct identification of regular liquid soap as an effective disinfectant. HPPs were 1.18 times more likely to be aware of regular liquid soap's efficacy compared with LPP (adjusted OR with HFMD experience 2.18, 99% CI 1.09–4.33, Table 4). Positive attitudes towards regular cleaning of high-contact surfaces (Q18) were also a facilitator for good practise of HFMD-PS; every unit increase in Q18 score increases odds of HPP by 8.33 times (99% CI 2.78–31.26, Supplementary Table 5). Nonetheless, this association becomes non-significant when adjusted for ethnicity and Q16 and Q17 scores (adjusted OR 1.95, 99% CI 0.42–8.95, Supplementary Table 5).

HPPs were also approximately three times as likely as LPPs to always wash their own hands or their children's hands according to proper handwashing steps (Q7 and Q8 adjusted OR 2.84 to 3.54, 99% CI 1.43 to 7.15, Supplementary Table 6). Other practises associated with high practise include the following: (1) cleaning children toys daily (Q9 adjusted OR 4.72, 99% CI 1.28-17.4, Supplementary Table 6) or (2) with warm water and detergent (Q11 adjusted OR 3.61, 99% CI 1.81-7.21, Supplementary Table 6), (3) the use of household bleach (Q13 adjusted OR 6.25 to 33.53, 99% CI 3.27 to 142.02, Supplementary Table 6), and (4) keeping the child at home until symptom resolution even if it is longer than the MC duration (Q14 adjusted OR 2.46, 99% CI 1.03–5.85, Supplementary Table 6). The question scores for all practises were all positively related to HPP status, suggesting that all questions were useful in assessing HFMD preventive practise levels amongst parents. The strongest association was observed in scores for questions regarding cleaning of toys (Q9, Q10, and Q11). These questions encompass the cleaning agent used (Q11 adjusted OR with HFMD experience 56.7, 99% CI 7.06–455.61, Supplementary Table 6), when the children's toys are cleaned in relation to playtime (Q10 adjusted OR with HFMD experience 52.36, 99% CI 7.54–363.55, Supplementary Table 6), and the frequency of cleaning daily, weekly, or monthly (Q9 adjusted OR with HFMD experience 20.36, 99% CI 4.72–87.83, Supplementary Table 6).

#### Teacher

Amongst teachers, being of Indian ethnicity (adjusted OR 4.43, 99% CI 1.03–19.04, Table 2) was the sole facilitator for higher practise levels of HFMD-PS, regardless of the centre's HFMD experience (adjusted OR with HFMD experience 4.65, 99% CI 1.07–20.26, Table 2).

## Discussion

This is the first study in Singapore to focus on understanding HFMD KAP levels amongst main caregivers, parents, and childcare centre teachers, of the most susceptible age group, i.e., children under 5 years amidst the ongoing COVID-19 pandemic. The respondents of this study displayed generally positive knowledge and attitudes towards HFMD preventive practises, albeit poor compliance with implementing preventive practises at home and in the childcare centres. This highlights potential gaps between knowledge and/or attitudes, and good hygiene practises for HFMD prevention. Similar gaps have also been observed in other studies assessing hand hygiene KAP to prevent HFMD or similar diseases such as diarrhoea in Singapore and Malaysia (11–13). However, studies in Thailand and China reported statistical significant correlations between knowledge and the practise of HFMD-PS, albeit in mixed directions (8–10). In a study from northern Thailand, moderate to high knowledge levels, respectively, increased odds of HFMD infection by 1.35 and 0.61 times (10). Nonetheless, it is important to note that these studies were conducted in relatively rural areas where respondents are generally with lower literacy levels and lower household income levels.

In the following subsections, we explore potential areas for consideration when formulating future prevention policies and strategies locally.

### Education and misconception

High awareness of touching contaminated surfaces as a potential source of HFMD transmission/infection, but not contact with stool, was observed in both parents and teachers from the high practise and low practise groups. This could have resulted from an emphasis on regular cleaning of high-contact surfaces in general guidelines against infectious diseases spread. Thus, touching surfaces contaminated by an infected case may have been mistaken as a potential mode of HFMD transmission, instead of actual residual contaminated body fluids, droplets, saliva, or stool left on the surface. In contrast, a significantly higher proportion of parents with children under 5 years in China were more likely to correctly identify stool as a transmission route (*n* = 316, 31.9%) compared with contact with contaminated surfaces (*n* = 266, 26.8%) (15). Nonetheless, proportions of parents with correct knowledge for identical HFMD transmission modes were consistently higher in this study.

Accurate knowledge of HFMD's infectious period was also low in all respondents, suggesting a critical need for more education efforts focusing on HFMD infectious period. A higher knowledge level of this may increase receptiveness and cooperation levels from parents and teachers in compliance towards preventive measures against HFMD. In particular, isolation of symptomatic children, postulated as the most effective measure in preventing centre outbreaks, may be enforced and supported to a higher degree by both parents and teachers after acquiring accurate knowledge regarding the disease's infectious period (16, 17).

Parents with lower education levels were observed to have lower levels of practising prevention measures at homes. A lower literacy level could have impeded comprehension of disseminated education materials to increase HFMD-related knowledge and prevention measures, resulting in their poorer adoption (18). Likewise, Saudi Arabian parents' literacy level was observed to be directly associated with their offspring's hand hygiene practise level in children (19). This suggests a potential need to intentionally review and simplify the complexity of current public engagement and education materials to enhance risk communication. Parents play an enormous role in inculcating their children with the correct hand hygiene practises, to instil long-term good hand hygiene practises in the next generation (15, 16). Sustained good hand hygiene has potentially long-term implications on preventing HFMD and other infectious diseases with similar transmission modes. Thus, it is important to improve current levels of HFMD-related knowledge and its prevention strategies, especially on good hand hygiene amongst parents with lower education levels.

### Hand hygiene practises

Despite majority acknowledging the importance and efficacy of following proper handwashing steps in protecting themselves or their children against infectious disease, parents and teachers did not always enforce compliance with proper handwashing steps. A low frequency of compliance in washing their children's hand as well as their own hands was prevalent amongst all parents, but more prominent amongst low practise parents. Teachers tend to be non-compliant when washing their own hands, but complied to proper handwashing steps more frequently when assisting the children with handwashing. This could be attributed to various reasons, including but not limited to (i) their professional capacity as early childhood educator doing their due diligence (20), (ii) the pertinence to adhere to infection prevention guidelines due to potentially increased transmission risk in a childcare centre, compared with at homes (17), and (iii) the motivation to avoid HFMD outbreaks due to the anticipated difficulties and/or additional burden resulting from a HFMD-related centre closure (as seen from teachers' experience with centre episodes or outbreaks in this study) (20).

The direct association between a proportion of respondents complying with proper handwashing steps and the compliance level to prevention measures was present in both groups, but especially amongst the parents. This suggests that strengthening proper handwashing practise amongst parents may also be effective in increasing compliance with prevention measures. Nonetheless, evidence has shown that solely washing hands with soap may not sufficiently protect children against HFMD (1). Following proper handwashing steps is as important as using the correct cleanser for effective handwashing (21). Given the high-risk setting of a childcare centre and the important role model of both teachers and parents in instilling good hand hygiene amongst the children (22, 23), more emphasis and outreach are needed to guide both parents and teachers.

### Cleaning and disinfection

Amongst the teachers in the high or low practise group, there was no significant difference in attitudes towards regular cleaning and disinfection of toys and high-contact surfaces, centre closures resulting from HFMD outbreaks, and acquiring more HFMD-related knowledge. This may be attributed to their awareness and acceptance for guidelines to creating and maintaining a safe environment in the centre, as part of their childhood educator's responsibility (20). Most Singaporean childcare teachers interviewed in a study explicitly mentioned negative feelings including those of guilt, worry, and stress, should a future outbreaks occur in their centres (20).

The risk of exposure to HFMD viruses is high in both school and home environments (17). Thus, good cleaning and disinfection practises should be consistently implemented in both environments to effectively protect children against HFMD. As the primary caregivers in these respective settings, parents and teachers play equally crucial roles in implementing or enforcing hygiene practises to advance this purpose.

Amongst parents, the strongest facilitator for high practise of prevention measures (in the knowledge and attitude domains) was the awareness of regular liquid soap as an effective disinfectant. Given liquid soap's high accessibility in Singapore's built environment, an increased emphasis on its efficacy as a disinfectant in education efforts could potentially increase practise levels at home. Within the practise domain, the strongest facilitators were the occasions for cleaning and the cleaning agent used for the children's toys. This further supports that increased awareness of regular liquid soap's efficacy in education materials may improve adoption levels of prevention measures. In addition, the study results indicate that parents who cleaned their children's toys at least before or after play time were more likely to be high adopters of prevention measures. This suggests the importance of emphasis on cleaning or sanitising toys before and after playtime, rather than the absolute frequency of cleaning, in outreach efforts to improve prevention practise levels at homes. This may be especially important as frequency of cleaning may be insufficiently protective against HFMD, if it is not practised at critical timings (1).

Amongst teachers, the prevention measure most associated with high adoption was a teacher's response to a failed health cheque in the centre. The role played by teachers to protect students from infectious diseases is ever pertinent. In childcare centres, teachers act as an effective first line of defence against HFMD transmission in this high-risk setting through high adoption levels of prevention practises. This is because most children in this age group have not yet developed the cognitive ability to consciously practise the recommended prevention measures. Sun et al. (24) showed that teachers had significantly more influence over a primary school student's handwashing habits before meals, and after using toilets or touching dogs, compared with family and peers. This suggests that promoting handwashing behaviours amongst students through teacher-involved participatory hygiene education may assist in preventing infectious disease transmission.

### Vaccine

To date, there are only three licenced monovalent enterovirus 71 vaccines offered in China (25). Given the numerous enteroviruses that can cause HFMD infection, an effective multi-valent vaccine remains elusive. Nonetheless, parent respondents in this study expressed positive vaccine acceptance, with a strong concern for vaccine safety and efficacy amongst those with neutral attitudes. Vaccine acceptance is influenced by the public's level of trust in the government, in addition to safety and efficacy (26–28). According to the Edelman Trust Barometer 2021 report, Singapore's government has high levels of public trust and is the most trusted institute in the country by respondents (29). Thus, the local health authorities' appraisal of any vaccine candidate's safety and efficacy prior to making it available in Singapore is likely to attribute to positive vaccine acceptance.

### Potential impact of COVID-19 on HFMD prevention

The adoption of non-pharmaceutical interventions (NPIs) in different populations during the COVID-19 pandemic is likely to decrease HFMD incidence. These NPIs generally advocated for increased personal hygiene and social distancing levels to protect oneself from COVID-19. There is a potential overlap in prevention measures against COVID-19 and HFMD, especially in terms of maintaining high levels of personal hygiene—hand and environmental hygiene—to prevent infection and transmission. Similar to Singapore, China reported a significantly lower annual HFMD incidence in 31 provincial capitals in 2020 (5, 30). The decreased incidence was strongly associated with childcare centre closures in these cities due to COVID-19 outbreaks (30). Compared with nine other common respiratory and gastrointestinal diseases reported in China in 2020, the decrease in HFMD incidence was the most pronounced (31). Thus, HFMD outbreaks are an unlikely cause of concern during the COVID-19 pandemic, especially during its peak when stricter measures against COVID-19 transmission were implemented (30). The above evidence clearly indicates the effectiveness of COVID-19 NPIs on mitigating HFMD transmission as HFMD incidence increased again in later 2020 when NPIs were more relaxed (30).

Frequent release of COVID-19 public health advisories and increased susceptibility to COVID-19 infection could have attributed to the similar knowledge and perception levels of HFMD prevention measures between high practise and low practise groups in this study. Studies have also demonstrated increased levels of knowledge and perceived importance of hand and environmental hygiene practises during the current and past pandemics involving respiratory illnesses (32–37). However, this may not always lead to increased practise levels (32–37). The increased practise levels were associated with high knowledge levels of self-protection behaviours, less negative perceptions of hand hygiene practises, self-efficacy, perceived susceptibility to COVID-19, and perceived severity of COVID-19 (32–35).

### Limitations

This study has a number of limitations, the most prominent being the cross-sectional nature preventing further assessment of temporal relationships between the exposure and outcome variables. Thus, causality cannot be established, while reverse causation still potentially exists in this study.

Second, the small study sample size could have skewed mean question scores and proportions, although these groups had comparable demographics (Table 1).

Third, the study attempted to account for confounding by adjusting for identified confounders, but possibility of residual confounding cannot be overlooked. For example, knowledge and attitude levels towards good hand hygiene practises amongst parents may have been confounded by the data collection period, i.e., during COVID-19 pandemic. Thus, the knowledge and attitudes towards hand hygiene practises and cleaning of surroundings expressed by study respondents could be inflated, especially when compared with pre-pandemic days (32–37). This limits the validity of our study findings to pandemic settings and may not reflect true levels or gaps in HFMD KAP amongst parents and teachers to guide future interventions.

Fourth, recall limitation and social desirability bias may be present as respondents, especially teachers, may be tempted to choose the ‘right' answer rather than the ‘true' answer despite anonymity. This is especially plausible for teacher respondents given their capacity as a professional and the inability to verify accuracy of responses from respondents regardless of parent or teacher.

Fifth, there may be selection bias due to the sampling method used—convenience sampling from a local childcare centre organisation. Most respondents in this study were from non-public childcare centres (parents 60.9%, teachers 70.8%), whereas the general Singapore population in 2018 only had approximately 40% children enrolled in private childcare centres (38). This suggests a potential skew in respondents towards the private childcare centres. This could be due to a higher proportion of (i) private childcare centres to non-private childcare centres owned by the organisation the sample was recruited through or (ii) childcare centres located in relatively upscale neighbourhoods where residents tend to enrol their children in the non-private childcare centres likely because of geographical convenience.

Lastly, this study utilised a self-developed questionnaire and scoring scheme to assess the KAP levels of parents and teachers. This approach limits the comparability of this study's results to similar ones performed in other countries as different assessment tools and frameworks were utilised. However, this also highlights the current lack of a validated and universal questionnaire to assess HFMD-related KAP amongst caregivers.

## Conclusion

In conclusion, this study found an existing gap between the positive levels of knowledge and attitudes of parents and teachers towards HFMD and actual prevention practise levels. Existing levels of knowledge and attitudes of parents and teachers were not high, and only a small proportion of respondents had a high level of prevention practises. Awareness of regular liquid soap's efficacy as a disinfectant, toy cleaning before and after playtime, and the compliance to frequent proper handwashing steps needs to be enforced amongst parents and teachers to reduce the risk of HFMD transmission, even amidst pandemics.

## Data availability statement

The raw data supporting the conclusions of this article will be made available by the authors, without undue reservation.

## Author contributions

JP contributed to the conception and design of the study and supervised the data collection and analysis. MW organised the database, performed the statistical analysis, prepared results visualisation, and wrote the original draught of the manuscript. All authors contributed to manuscript revision, read, and approved the submitted version.

## Funding

The research work was funded by Saw Swee Hock School of Public Health, National University of Singapore (NUS ODPRT—Reimagine Research Grant, grant number A-0006299-00-00 and A-0006299-01-00). The funder of the study had no role in study design, data collection, data analysis, data interpretation, or writing of the report.

## Conflict of interest

The authors declare that the research was conducted in the absence of any commercial or financial relationships that could be construed as a potential conflict of interest.

## Publisher's note

All claims expressed in this article are solely those of the authors and do not necessarily represent those of their affiliated organizations, or those of the publisher, the editors and the reviewers. Any product that may be evaluated in this article, or claim that may be made by its manufacturer, is not guaranteed or endorsed by the publisher.
