# Supplementary material for: The knowledge, attitudes and practices of hand, foot, and mouth disease prevention strategies amongst parents and educators of children under 5 years amidst COVID-19 pandemic: A cross-sectional study
Source: Front Public Health. 2022 Oct 17;10:908004. doi: 10.3389/fpubh.2022.908004 (PMC9619192; doi:10.3389/fpubh.2022.908004)
Supplement: Supplementary file 2 [file Table_2.docx]

# Table 5. Attitudes of parents and teachers towards HFMD-PS

| **Attitude** | | **Number of Respondents (%)** | | | **p** | **Crude OR (99% Cl)** | **Adjusted OR ^a^ (99% CI)** | **Adjusted OR ^b^ (99% CI)** |
| --- | --- | --- | --- | --- | --- | --- | --- | --- |
| **Question** | **Response options** | **Overall** | **High Practise** | **Low Practise** |  |  |  |  |
| **Parents** | | **N=404** | **N=99** | **N=305** |  |  |  |  |
| Overall Score | Mean (SD) | 3.03 (2.02) () | 3.80 (1.63) () | 2.78 (2.07) () | <0.01 | 1.32 (1.12-1.56) | 0.97 (0.77-1.23) | 0.94 (0.74-1.20) |
|  | Median (IQR) | 3.17 (1.67-4.5); Range: -4.08-6.5 | 4.08 (2.5-4.67); Range: 0.17-6.5 | 2.92 (1.25-4.33); Range: -4.08-6.5 | <0.01 | NA | | |
| 15 | Time-consuming | 116 (28.71) | 19 (19.19) | 97 (31.8) | 0.02 | 0.51 (0.25-1.06)ᶧ | 0.98 (0.43-2.24) | 0.91 (0.40-2.11) |
|  | Important | 277 (68.56) | 79 (79.8) | 198 (64.92) | <0.01 | **2.13 (1.04-4.36)*** | 0.95 (0.39-2.29) | 1.04 (0.42-2.54) |
|  | Tedious | 86 (21.29) | 10 (10.1) | 76 (24.92) | <0.01 | **0.34 (0.13-0.85)*** | 0.70 (0.24-2.03) | 0.70 (0.24-2.05) |
|  | Excessive | 26 (6.44) | 4 (4.04) | 22 (7.21) | 0.35 | 0.54 (0.13-2.27) | 1.62 (0.32-8.20) | 1.60 (0.31-8.18) |
|  | Protective against infectious diseases | 259 (64.11) | 72 (72.73) | 187 (61.31) | 0.04 | 1.68 (0.87-3.24)ᶧ | 0.54 (0.22-1.32) | 0.49 (0.20-1.21)ᶧ |
|  | Others | 2 (0.5) | 1 (1.01) | 1 (0.33) | 0.43 | 3.10 (0.08-119.95) | 2.34 (0.05-100.11) | 1.83 (0.04-78.78) |
|  | Q15 Score: Mean (SD) | 0.52 (0.47) | 0.68 (0.35) | 0.47 (0.50) | <0.01 | **2.95 (1.39-6.25)*** | 0.58 (0.17-1.97) | 0.61 (0.18-2.08) |
| 16 | Time-consuming | 99 (24.5) | 14 (14.14) | 85 (27.87) | <0.01 | **0.43 (0.19-0.96)*** | 0.86 (0.32-2.31) | 0.83 (0.31-2.21) |
|  | Important | 280 (69.31) | 81 (81.82) | 199 (65.25) | <0.01 | **2.40 (1.14-5.02)*** | 0.78 (0.27-2.27) | 0.85 (0.29-2.49) |
|  | Tedious | 104 (25.74) | 11 (11.11) | 93 (30.49) | <0.01 | **0.28 (0.12-0.69)*** | 0.55 (0.19-1.59) | 0.52 (0.18-1.51) |
|  | Excessive | 19 (4.7) | 3 (3.03) | 16 (5.25) | 0.58 | 0.56 (0.11-2.94) | 1.48 (0.23-9.63) | 1.39 (0.20-9.43) |
|  | Protective against infectious diseases | 243 (60.15) | 73 (73.74) | 170 (55.74) | <0.01 | **2.23 (1.15-4.31)*** | 0.86 (0.33-2.28) | 0.75 (0.28-2.02) |
|  | Others | 8 (1.98) | 0 (0) | 8 (2.62) | 0.21 | Omitted | Omitted | Omitted |
|  | Q16 Score: Mean (SD) | 0.51 (0.47) | 0.71 (0.34) | 0.44 (0.48) | <0.01 | **4.43 (1.98-9.94)*** | NA | |
| 17 | Time-consuming | 141 (34.9) | 19 (19.19) | 122 (40) | <0.01 | **0.36 (0.17-0.73)*** | 0.60 (0.24-1.51) | 0.58 (0.23-1.47) |
|  | Important | 254 (62.87) | 76 (76.77) | 178 (58.36) | <0.01 | **2.36 (1.19-4.66)*** | 0.67 (0.25-1.78) | 0.70 (0.26-1.86) |
|  | Tedious | 106 (26.24) | 13 (13.13) | 93 (30.49) | <0.01 | **0.34 (0.15-0.79)*** | 0.84 (0.30-2.34) | 0.89 (0.32-2.51) |
|  | Excessive | 33 (8.17) | 3 (3.03) | 30 (9.84) | <0.01 | 0.29 (0.06-1.40)ᶧ | 1.45 (0.22-9.52) | 1.41 (0.21-9.49) |
|  | Protective against infectious diseases | 216 (53.47) | 68 (68.69) | 148 (48.52) | <0.01 | **2.33 (1.24-4.38)*** | 0.87 (0.35-2.12) | 0.86 (0.35-2.12) |
|  | Others | 10 (2.48) | 1 (1.01) | 9 (2.95) | 0.46 | 0.34 (0.02-5.15) | 0.77 (0.05-13.05) | 0.71 (0.04-12.43) |
|  | Q17 Score: Mean (SD) | 0.21 (0.39) | 0.40 (0.30) | 0.15 (0.40) | <0.01 | **6.99 (2.66-18.36)*** | NA | |
| 18 | Time-consuming | 102 (25.25) | 11 (11.11) | 91 (29.84) | <0.01 | **0.29 (0.12-0.71)*** | 0.47 (0.18-1.20)ᶧ | 0.45 (0.18-1.17)ᶧ |
|  | Important | 303 (75) | 82 (82.83) | 221 (72.46) | 0.05 | 1.83 (0.86-3.93)ᶧ | 0.64 (0.26-1.61) | 0.71 (0.28-1.82) |
|  | Tedious | 77 (19.06) | 6 (6.06) | 71 (23.28) | <0.01 | **0.21 (0.07-0.66)*** | 0.39 (0.12-1.34) | 0.40 (0.17-1.36) |
|  | Excessive | 21 (5.2) | 1 (1.01) | 20 (6.56) | 0.03 | 0.15 (0.01-2.07) | 0.34 (0.03-5.48) | 0.32 (0.02-5.55) |
|  | Protective against infectious diseases | 248 (61.39) | 74 (74.75) | 174 (57.05) | 0.002 | **2.23 (1.14-4.34)*** | 0.98 (0.43-2.25) | 0.92 (0.40-2.12) |
|  | Others | 4 (0.99) | 0 (0) | 4 (1.31) | 0.58 | Omitted | Omitted | Omitted |
|  | Q18 Score: Mean (SD) | 0.33 (0.35) | 0.48 (0.21) | 0.28 (0.37) | <0.01 | **9.33 (2.78-31.26)*** | 1.95 (0.42-8.95) | 2.03 (0.43-9.56) |
| 19a | Reasonable | 274 (67.82) | 74 (74.75) | 200 (65.57) | 0.11 | 1.55 (0.79-3.04) | 1.11 (0.53-2.34) | 1.08 (0.51-2.28) |
|  | Too long | 136 (33.66) | 27 (27.27) | 109 (35.74) | 0.14 | 0.67 (0.35-1.30) | 0.95 (0.46-1.96) | 0.99 (0.48-2.05) |
|  | Too short | 6 (1.49) | 2 (2.02) | 4 (1.31) | 0.64 | 1.55 (0.16-14.73) | 1.40 (0.09-24.19) | 1.36 (0.09-21.10) |
|  | Q19a Score: Mean (SD) | 0.01 (0.70) | 0.11 (0.66) | -0.02 (0.71) | 0.09 | 1.33 (0.85-2.08) | 1.05 (0.64-1.72) | 1.03 (0.63-1.69) |
| 19b | Can help to stop HFMD spread | 311 (76.98) | 80 (80.81) | 231 (75.74) | 0.34 | 1.35 (0.64-2.83) | 1.21 (0.54-2.75) | 1.13 (0.50-2.59) |
|  | Is not necessary | 19 (4.7) | 3 (3.03) | 16 (5.25) | 0.58 | 0.56 (0.11-2.94) | 0.64 (0.11-3.68) | 0.74 (0.13-4.30) |
|  | Will be too much of a burden to me | 107 (26.49) | 12 (12.12) | 95 (31.15) | <0.01 | **0.30 (0.13-0.72)*** | **0.38 (0.15-0.94)*** | 0.40 (0.16-0.998)ᶧ |
|  | Poses inconvenience but I can make appropriate arrangements to cope | 220 (54.46) | 45 (45.45) | 175 (57.38) | 0.05 | 0.62 (0.34-1.13)ᶧ | 0.67 (0.34-1.30) | 0.65 (0.33-1.26) |
|  | Others | 3 (0.74) | 0 (0) | 3 (0.98) | 1 | Omitted | Omitted | Omitted |
|  | Q19b Score: Mean (SD) | 0.33 (0.33) | 0.37 (0.27) | 0.32 (0.35) | 0.15 | 1.59 (0.62-4.09) | 1.41 (0.48-4.18) | 1.25 (0.42-3.76) |
| 20 | Yes | 264 (65.35) | 68 (68.69) | 196 (64.26) | 0.77 | 1 | 1 | 1 |
|  | No | 10 (2.48) | 2 (2.02) | 8 (2.62) |  | 0.72 (0.09-5.70) | 1.07 (0.11-10.04) | 1.05 (0.10-10.85) |
|  | Depends | 130 (32.18) | 29 (29.29) | 101 (33.11) |  | 0.83 (0.43-1.59) | 1.05 (0.51-2.17) | 1.06 (0.51-2.20) |
|  | Q20 Score: Mean (SD) | 0.95 (0.31) | 0.96 (0.28) | 0.95 (0.32) | 0.72 | 1.14 (0.41-3.20) | 0.97 (0.32-2.96) | 0.99 (0.31-3.16) |
| 20 (Depends) | Cost | 37 (28.46) | 8 (27.59) | 29 (28.71) | 1 | 0.95 (0.29-3.17) | 0.83 (0.19-3.63) | 0.81 (0.17-3.79) |
|  | Safety | 99 (76.15) | 21 (72.41) | 78 (77.23) | 0.63 | 0.77 (0.23-2.65) | 1.14 (0.23-5.74) | 1.28 (0.23-7.09) |
|  | Insurance coverage | 30 (23.08) | 5 (17.24) | 25 (24.75) | 0.46 | 0.63 (0.16-2.56) | 0.80 (0.16-3.98) | 0.67 (0.13-3.52) |
|  | Number of doses | 47 (36.15) | 8 (27.59) | 39 (38.61) | 0.38 | 0.61 (0.18-2.00) | 0.37 (0.08-1.60) | 0.40 (0.09-1.87) |
|  | Vaccine efficacy | 107 (82.31) | 24 (82.76) | 83 (82.18) | 1 | 1.04 (0.25-4.36) | 0.79 (0.14-4.46) | 0.75 (0.12-4.57) |
|  | Doctor's recommendation | 68 (52.31) | 13 (44.83) | 55 (54.46) | 0.40 | 0.68 (0.23-2.02) | 0.38 (0.09-1.60) | 0.46 (0.10-2.04) |
|  | Child's age | 65 (50) | 14 (48.28) | 51 (50.5) | 1 | 0.92 (0.31-2.71) | 0.60 (0.16-2.32) | 0.79 (0.19-3.30) |
|  | Risk of HFMD infection | 62 (47.69) | 15 (51.72) | 47 (46.53) | 0.68 | 1.23 (0.42-3.65) | 0.77 (0.19-3.11) | 0.63 (0.15-2.71) |
|  | Risk of HFMD reinfection | 55 (42.31) | 15 (51.72) | 40 (39.6) | 0.29 | 1.63 (0.55-4.87) | 1.14 (0.28-4.59) | 4.67 (0.35-6.51) |
|  | Others | 5 (3.85) | 0 (0) | 5 (4.95) | 0.59 | Omitted | Omitted | Omitted |
| 21 | Yes | 235 (58.17) | 54 (54.55) | 181 (59.34) | 0.41 | 1 | 1 | 1 |
|  | No | 169 (41.83) | 45 (45.45) | 124 (40.66) |  | 1.22 (0.67-2.22) | 1.23 (0.63-2.38) | 1.33 (0.68-2.63) |
|  | Q21 Score: Mean (SD) | 0.16 (0.99) | 0.09 (1.00) | 0.19 (0.98) | 0.41 | 0.91 (0.67-1.22) | 0.90 (0.65-1.26) | 0.87 (0.62-1.21) |
| 21 (Yes) | Brochure | 144 (61.28) | 33 (61.11) | 111 (61.33) | 1 | 0.99 (0.44-2.25) | 1.05 (0.42-2.58) | 1.00 (0.40-2.49) |
|  | Talk by HCP | 61 (25.96) | 17 (31.48) | 44 (24.31) | 0.29 | 1.43 (0.60-3.44) | 1.55 (0.59-4.04) | 1.60 (0.61-4.21) |
|  | Poster | 63 (26.81) | 13 (24.07) | 50 (27.62) | 0.73 | 0.83 (0.33-2.09) | 0.83 (0.31-2.24) | 0.87 (0.32-2.37) |
|  | Social media | 159 (67.66) | 35 (64.81) | 124 (68.51) | 0.62 | 0.85 (0.36-1.97) | 0.71 (0.28-1.76) | 0.74 (0.29-1.86) |
|  | Others | 11 (4.68) | 2 (3.7) | 9 (4.97) | 1 | 0.74 (0.09-5.74) | 0.98 (0.17-8.22) | 0.99 (0.12-8.32) |
| **Teachers** | | **N=240** | **N=28** | **N=212** |  |  |  |  |
| Overall Score | Mean (SD) | 3.42 (1.67) | 3.68 (1.41) | 3.39 (1.71) | 0.33 | 1.12 (0.80-1.55) | 1.13 (0.80-1.60) | 1.15 (0.81-1.63) |
|  | Median (IQR) | 3.88 (2.17-4.83); Range: -3.75-5.33 | 4.13 (2.46-5.04); Range: 0.5-5.33 | 3.54 (2.17-4.83); Range: -3.75-5.33 | 0.59 | NA | | |
| 8 | Time-consuming | 61 (25.42) | 4 (14.29) | 57 (26.89) | 0.17 | 0.45 (0.11-1.93) | 0.39 (0.08-1.90) | 0.37 (0.07-1.83) |
|  | Important | 198 (82.5) | 23 (82.14) | 175 (82.55) | 1 | 0.97 (0.25-3.77) | 1.18 (0.27-5.19) | 1.17 (0.27-5.16) |
|  | Tedious | 29 (12.08) | 4 (14.29) | 25 (11.79) | 0.76 | 1.25 (0.28-5.56) | 1.48 (0.31-6.98) | 1.44 (0.30-6.83) |
|  | Excessive | 13 (5.42) | 3 (10.71) | 10 (4.72) | 0.18 | 2.42 (0.41-14.39) | 2.32 (0.29-18.77) | 2.00 (0.21-18.89) |
|  | Protective against infectious diseases | 170 (70.83) | 21 (75) | 149 (70.28) | 0.67 | 1.27 (0.39-4.17) | 1.64 (0.46-5.88) | 1.78 (0.48-6.51) |
|  | Others | 3 (1.25) | 1 (3.57) | 2 (0.94) | 0.31 | 3.89 (0.16-95.25) | 2.74 (0.10-74.95) | 2.84 (0.10-78.33) |
|  | Q8 Score: Mean (SD) | 0.66 (0.40) | 0.69 (0.37) | 0.66 (0.4) | 0.67 | 1.23 (0.32-4.81) | 1.71 (0.37-7.84) | 1.87 (0.39-8.84) |
| 10 | Time-consuming / Tedious / Excessive | 37 (15.42) | 2 (7.14) | 35 (16.51) | 0.43 | 1 | 1 | 1 |
|  | Important / Protective against infectious diseases | 201 (83.75) | 26 (92.86) | 175 (82.55) |  | 2.60 (0.37-18.26) | 4.84 (0.45-51.58) | 5.19 (0.48-56.44) |
|  | Others | 2 (0.83) | 0 (0) | 2 (0.94) |  | Omitted | Omitted | Omitted |
|  | Q10 Score: Mean (SD) | 0.68 (0.73) | 0.86 (0.52) | 0.66 (0.75) | 0.08 | 1.65 (0.78-3.48) | 2.23 (0.91-5.47) | 2.30 (0.93-5.70) |
| 13 | Time-consuming | 25 (10.42) | 2 (7.14) | 23 (10.85) | 0.75 | 0.63 (0.14-2.84) | 0.7 (0.15-3.26) | 0.71 (0.15-3.27) |
|  | Important | 206 (85.83) | 21 (75) | 185 (87.26) | 0.09 | 0.44 (0.17-1.13) | 0.47 (0.18-1.26) | 0.49 (0.18-1.31) |
|  | Tedious | 21 (8.75) | 2 (7.14) | 19 (8.96) | 1 | 0.78 (0.17-3.55) | 0.85 (0.18-4.01) | 0.89 (0.19-4.2) |
|  | Excessive | 4 (1.67) | 0 (0) | 4 (1.89) | 1 | Omitted | Omitted | Omitted |
|  | Protective against infectious diseases | 181 (75.42) | 19 (67.86) | 162 (76.42) | 0.35 | 0.65 (0.28-1.53) | 0.58 (0.24-1.41) | 0.58 (0.24-1.42) |
|  | Others | 1 (0.42) | 0 (0) | 1 (0.47) | 1 | Omitted | Omitted | Omitted |
|  | Q13 Score: Mean (SD) | 0.48 (0.23) | 0.44 (0.27) | 0.49 (0.22) | 0.33 | 0.39 (0.05-3.30) | 0.31 (0.03-2.93) | 0.32 (0.03-3.01) |
| 14 | Time-consuming | 26 (10.83) | 2 (7.14) | 24 (11.32) | 0.75 | 0.60 (0.08-4.32) | 0.64 (0.09-4.78) | 0.66 (0.09-5.00) |
|  | Important | 205 (85.42) | 20 (71.43) | 185 (87.26) | 0.04 | 0.36 (0.11-1.21)ᶧ | 0.37 (0.11-1.28)ᶧ | 0.34 (0.10-1.22)ᶧ |
|  | Tedious | 24 (10) | 0 (0) | 24 (11.32) | 0.09 | Omitted | Omitted | Omitted |
|  | Excessive | 7 (2.92) | 0 (0) | 7 (3.3) | 1 | Omitted | Omitted | Omitted |
|  | Protective against infectious diseases | 181 (75.42) | 22 (78.57) | 159 (75) | 0.82 | 1.22 (0.35-4.29) | 1.23 (0.34-4.48) | 1.27 (0.35-4.63) |
|  | Others | 0 (0) | 0 (0) | 0 (0) | N/A | NA | NA | NA |
|  | Q14 Score: Mean (SD) | 0.48 (0.22) | 0.48 (0.19) | 0.48 (0.23) | 0.89 | 1.11 (0.11-11.38) | 0.97 (0.09-10.68) | 0.93 (0.08-10.33) |
|  | Too long | 17 (7.08) | 1 (3.57) | 16 (7.55) | 0.70 | 1 | 1 | 1 |
|  | Too short / Reasonable | 223 (92.92) | 27 (96.43) | 196 (92.45) |  | 2.20 (0.15-33.03) | 1.87 (0.12-28.74) | 2.02 (0.13-31.77) |
|  | Q21 Score: Mean (SD) | 0.86 (0.51) | 0.93 (0.38) | 0.85 (0.53) | 0.33 | 1.48 (0.38-5.75) | 1.37 (0.35-5.36) | 1.42 (0.36-5.64) |
| 27 | Yes | 152 (63.33) | 18 (64.29) | 134 (63.21) | 1 | 1.05 (0.36-3.09) | 0.93 (0.30-2.85) | 0.92 (0.30-2.83) |
|  | No | 88 (36.67) | 10 (35.71) | 78 (36.79) |  | 1 | 1 | 1 |
|  | Q27 Score: Mean (SD) | 0.27 (0.97) | 0.29 (0.98) | 0.26 (0.97) | 0.91 | 1.02 (0.6-1.76) | 0.96 (0.55-1.69) | 0.96 (0.55-1.68) |
| 27 (Yes) | Brochure | 25 (16.45) | 2 (11.11) | 23 (17.16) | 0.94 | 1 | 1 | 1 |
|  | Talk by HCP | 59 (38.82) | 7 (38.89) | 52 (38.81) |  | 1.55 (0.18-13.47) | 1.43 (0.16-13.2) | 1.44 (0.16-13.32) |
|  | Poster | 9 (5.92) | 1 (5.56) | 8 (5.97) |  | 1.44 (0.05-40.05) | 1.14 (0.02-55.53) | 1.17 (0.02-57.91) |
|  | Social media | 59 (38.82) | 8 (44.44) | 51 (38.06) |  | 1.80 (0.21-15.28) | 2.05 (0.23-18.06) | 2.08 (0.24-18.38) |
|  | Others | 0 (0) | 0 (0) | 0 (0) |  | NA | | |

OR, Odds ratio; *p<0.01; ᶧ0.01≤p<0.05; NA, Not Applicable; p, Fisher’s exact test p-value (categorical variable) or t-test p-value (continuous variable)
All **bolded** odds ratios indicate statistically significance results i.e. p<0.01 ^a^ Parents: adjusted for ethnicity, Q16 Score, Q17 Score; Teachers: adjusted for Q6 Score, Q20 Score
^b^ Parents: adjusted for ethnicity, Q16 Score, Q17 Score, HFMD Status (Yes as reference group); Teachers: adjusted for Q6 Score, Q20 Score, HFMD-related centre closure experience (Yes as reference group)
