# Supplementary material for: The knowledge, attitudes and practices of hand, foot, and mouth disease prevention strategies amongst parents and educators of children under 5 years amidst COVID-19 pandemic: A cross-sectional study
Source: Front Public Health. 2022 Oct 17;10:908004. doi: 10.3389/fpubh.2022.908004 (PMC9619192; doi:10.3389/fpubh.2022.908004)
Supplement: Supplementary file 3 [file Table_3.docx]

# Table 6. Practises of parents and teachers towards HFMD-PS

| **Practice** | | **Number of Respondents (%)** | | | **p** | **Crude OR (99% Cl)** | **Adjusted OR ^a^ (99% CI)** | **Adjusted OR ^b^ (99% CI)** |
| --- | --- | --- | --- | --- | --- | --- | --- | --- |
| **Question** | **Response options** | **Overall** | **High Practise** | **Low Practise** |  |  |  |  |
| **Parents** | | **N=404** | **N=99** | **N=305** |  |  |  |  |
| **Overall Score** | Mean (SD) | 2.82 (2.13) | 5.13 (0.70) | 2.06 (1.88) | <0.01 | NA | | |
|  | Median (IQR) | 3.23 (1.76-4.35); Range: -6.25-7.25) | 4.88 (4.63-5.53); Range: 4.37-7.25 | 2.54 (1.2-3.58); Range: -6.25-4.35) | <0.01 | NA | | |
| 7 | Not Always | 275 (68.07) | 41 (41.41) | 234 (76.72) | <0.01 | 1 | 1 | 1 |
|  | Always | 129 (31.93) | 58 (58.59) | 71 (23.28) |  | **4.66 (2.48-8.76)*** | **3.54 (1.76-7.15)*** | **3.50 (1.72-7.09)*** |
|  | Q7 Score: Mean (SD) | 0.53 (0.52) | 0.79 (0.25) | 0.44 (0.56) | <0.01 | **16.45 (4.83-55.97)*** | **11.30 (2.94-43.75)*** | **11.06 (2.85-42.98)*** |
| 8 | Not Always | 277 (68.73) | 44 (44.44) | 233 (76.64) | <0.01 | 1 | 1 | 1 |
|  | Always | 126 (31.27) | 55 (55.56) | 71 (23.36) |  | **4.1 (2.19-7.68)*** | **2.84 (1.43-5.65)*** | **3.1 (1.53-6.25)*** |
|  | Q8 Score: Mean (SD) | 0.50 (0.56) | 0.78 (0.25) | 0.41 (0.60) | <0.01 | **12.49 (3.84-40.54)*** | **7.65 (2.17-26.90)*** | **8.92 (2.45-32.51)*** |
| 9 | Not Everyday | 383 (94.8) | 86 (86.87) | 297 (97.38) | <0.01 | 1 | 1 | 1 |
|  | Everyday | 21 (5.2) | 13 (13.13) | 8 (2.62) |  | **5.61 (1.69-18.63)*** | **4.72 (1.28-17.4)*** | **4.31 (1.16-15.98)*** |
|  | Q9 Score: Mean (SD) | 0.33 (0.49) | 0.61 (0.24) | 0.24 (0.52) | <0.01 | **27.47 (7.36-102.46)*** | **23.40 (5.41-101.23)*** | **20.36 (4.72-87.83)*** |
| 10 | Before and after play time/Only after play time | 217 (53.71) | 73 (73.74) | 144 (47.21) | <0.01 | 1.86 x 10^7^ (0-NE) | 2.29 x 10^7^ (0-NE) | 1.89 x 10^7^ (0-NE) |
|  | Only before play time/Others | 128 (31.68) | 26 (26.26) | 102 (33.44) |  | 9.35 x 10^6^ (0-Not NE) | 1.29 x 10^7^ (0-NE) | 1.02 x 10^7^ (0-NE) |
|  | Never | 59 (14.6) | 0 (0) | 59 (19.34) |  | 1 | 1 | 1 |
|  | Q10 Score: Mean (SD) | 0.29 (0.57) | 0.63 (0.24) | 0.18 (0.60) | <0.01 | **67.50 (10.86-419.35)*** | **48.61 (7.20-328.11)*** | **52.36 (7.54-363.55)*** |
| 11 | Dry wipe | 50 (12.38) | 16 (16.16) | 34 (11.15) | 0.22 | 1.54 (0.66-3.58) | 1.25 (0.49-3.18) | 1.29 (0.50-3.32) |
|  | Wipe with wet cloth | 202 (50) | 48 (48.48) | 154 (50.49) | 0.82 | 0.92 (0.51-1.67) | 0.98 (0.51-1.90) | 0.10 (0.51-1.94) |
|  | Cleaned with detergent (soap) | 127 (31.44) | 41 (41.41) | 86 (28.2) | 0.02 | 1.80 (0.97-3.34)ᶧ | 1.57 (0.79-3.09)ᶧ | 1.60 (0.80-3.17) |
|  | Cleaned with warm water and detergent (soap) | 150 (37.13) | 63 (63.64) | 87 (28.52) | <0.01 | **4.39 (2.33-8.23)*** | **3.61 (1.81-7.21)*** | **3.88 (1.91-7.86)*** |
|  | Cleaned with household bleach | 21 (5.2) | 10 (10.1) | 11 (3.61) | 0.02 | 3.00 (0.93-9.65)ᶧ | 1.85 (0.50-6.91)ᶧ | 2.02 (0.53-7.62) |
|  | Not cleaned | 35 (8.66) | 0 (0) | 35 (11.48) | <0.01 | Omitted | Omitted | Omitted |
|  | Others | 54 (13.37) | 12 (12.12) | 42 (13.77) | 0.74 | 0.86 (0.35-2.13) | 0.90 (0.34-2.39) | 0.91 (0.34-2.45) |
|  | Q11 Score: Mean (SD) | 0.12 (0.38) | 0.32 (0.17) | 0.06 (0.41) | <0.01 | **69.97 (10.53-465.10)*** | **44.52 (5.71-347.44)*** | **56.70 (7.06-455.61)*** |
| 11 (Others) | Others (Disinfectant) | 3 (5.56) | 1 (8.33) | 2 (4.76) | 0.10 | 3.30 x 10^6^ (0-NE) | 5.20 X10^6^ (0-NE) | 1.26 x 10^8^ (0-NE) |
|  | Others (Sanitiser) | 28 (51.85) | 10 (83.33) | 18 (42.86) |  | 3.67 x 10^6^ (0-NE) | 6.92 X10^5^ (0-NE) | 4.39 x 10^6^ (0-NE) |
|  | Others (Water) | 8 (14.81) | 0 (0) | 8 (19.05) |  | Omitted | Omitted | Omitted |
|  | Others (Alcohol) | 13 (24.07) | 1 (8.33) | 12 (28.59) |  | 13.22 (0-NE) | 4.623 x 10^4^ (0-NE) | 1.18 x 10^5^ (0-NE) |
|  | Others (Others) | 2 (3.7) | 0 (0) | 2 (4.76) |  | 1 | 1 | 1 |
| 12 | Dry wipe | 5 (1.24) | 0 (0) | 5 (1.64) | <0.01 | Omitted | Omitted | Omitted |
|  | Wipe with wet mop/cloth | 102 (25.25) | 4 (4.04) | 98 (32.13) |  | 1.57 x 10^4^ (0-NE) | 3.61 x 10^3^ (0-NE) | 9.38 x 10^3^ (0-NE) |
|  | Cleaned with detergent (soap) | 129 (31.93) | 25 (25.25) | 104 (34.1) |  | 9.27 x 10^4^ (0-NE) | 2.23 x10^4^ (0-NE) | 5.90 x 10^4^ (0-NE) |
|  | Cleaned with warm water and detergent (soap) | 93 (23.02) | 33 (33.33) | 60 (19.67) |  | 2.12 x 10^5^ (0-NE) | 5.09 x 10^4^ (0-NE) | 1.46 x 10^5^ (0-NE) |
|  | Cleaned with household bleach | 49 (12.13) | 26 (26.26) | 23 (7.54) |  | 4.36 x 10^5^ (0-NE) | 1.05 x 10^5^ (0-NE) | 2.96 x 10^5^ (0-NE) |
|  | Not cleaned | 1 (0.25) | 0 (0) | 1 (0.33) |  | 1 | 1 | 1 |
|  | Others | 25 (6.19) | 11 (11.11) | 14 (4.59) |  | 3.03 x 10^5^ (0-NE) | 6.05 x 10^4^ (0-NE) | 1.67 x 10^5^ (0-NE) |
|  | Q12 Score: Mean (SD) | 0.73 (0.45) | 0.96 (0.20) | 0.65 (0.49) | <0.01 | **12.73 (3.3-49.11)*** | **13.17 (3.29-52.74)*** | **13.46 (3.35-54.14)*** |
| 12 (Others) | Others (Disinfectant) | 13 (54.17) | 7 (63.64) | 6 (46.15) | 0.52 | 8.64 x 10^6 (0-NE) | Model convergence not achieved | Model convergence not achieved |
|  | Others (Sanitiser) | 8 (33.33) | 3 (27.27) | 5 (38.46) |  | 4.44 x 10^6 (0-NE) | NA | NA |
|  | Others (Home mixture) | 1 (4.17) | 1 (9.09) | 0 (0) |  | Omitted | NA | NA |
|  | Others (Steam) | 2 (8.33) | 0 (0) | 2 (15.38) |  | 1 | NA | NA |
| 13 | Never | 167 (41.34) | 5 (5.05) | 162 (53.11) | <0.01 | 1 | 1 | 1 |
|  | ≥Once a week | 82 (20.3) | 45 (45.45) | 37 (12.13) |  | **39.41 (10.72-144.85)*** | 33.53 (8.67-129.72) | 31.45 (8.09-122.32) |
|  | ≥Once a month | 103 (25.5) | 34 (34.34) | 69 (22.62) |  | **15.97 (4.40-57.89)*** | 12.46 (3.27-47.44) | 11.81 (3.08-45.20) |
|  | When someone in the household is sick | 35 (8.66) | 12 (12.12) | 23 (7.54) |  | **16.90 (3.82-74.73)*** | 28.40 (5.68-142.02) | 28.35 (5.65-142.08) |
|  | Others | 17 (4.21) | 3 (3.03) | 14 (4.59) |  | 6.94 (0.93-51.99)ᶧ | 6.25 (0.78-50.44)ᶧ | 5.98 (0.74-48.18)ᶧ |
|  | Q13 Score: Mean (SD) | -0.10 (0.79) | 0.52 (0.46) | -0.31 (0.76) | <0.01 | **6.25 (3.37-11.55)*** | 7.6 (3.64-15.88) | 7.43 (3.54-15.59) |
| 14 | Inform CC immediately | 390 (96.53) | 98 (98.99) | 292 (95.74) | 0.20 | 4.36 (0.30-64.27) | 4.54 (2.28-74.64) | 4.81 (0.29-78.67) |
|  | Keep child at home until MC expiration | 303 (75) | 72 (72.73) | 231 (75.74) | 0.59 | 0.85 (0.43-1.68) | 0.79 (0.37-1.69) | 0.81 (0.38-1.73) |
|  | Keep child at home until all symptoms disappear, even if shorter than MC duration | 115 (28.47) | 38 (38.38) | 77 (25.25) | 0.02 | 1.84 (0.98-3.47)ᶧ | 1.74 (0.86-3.52)ᶧ | 1.76 (0.87-3.59)ᶧ |
|  | Keep child at home until all symptoms disappear, even if longer than MC duration | 296 (73.27) | 84 (84.85) | 212 (69.51) | <0.01 | **2.46 (1.12-5.41)*** | **2.46 (1.03-5.85)*** | 2.33 (0.97-5.61)ᶧ |
|  | Bring child back to CC once they feel better, even with persisting symptoms | 10 (2.48) | 0 (0) | 10 (3.28) | 0.128 | Omitted | Omitted | Omitted |
|  | Q14 Score: Mean (SD) | 0.43 (0.29) | 0.52 (0.18) | 0.40 (0.31) | <0.01 | **13.69 (2.49-75.41)*** | **13.29 (2.24-78.74)*** | **12.74 (2.08-78.04)*** |
| **Teachers** | | **N=240** | **N=28** | **N=212** |  |  |  |  |
| Overall Score | Mean (SD) | 3.03 (1.21) | 6.76 (0.15) | 5.44 (1.11) | <0.01 | NA | NA | NA |
|  | Median (IQR) | 2.67 (2.17-3.92); Range: 0.92-6.67 | 6.67 (6.67-6.92); Range: 6.67-7.25 | 5.92 (5.17-6.17); Range: -0.25-6.42 | <0.01 | NA | NA | NA |
| 7 | Not Always | 98 (40.83) | 14 (50) | 84 (39.62) | 0.31 | 1 | 1 | 1 |
|  | Always | 142 (59.17) | 14 (50) | 128 (60.38) |  | 0.66 (0.23-1.85) | 0.84 (0.28-2.48) | 0.85 (0.28-2.52) |
|  | Q7 Score: Mean (SD) | 0.78 (0.29) | 1 (0) | 0.75 (0.3) | <0.01 | Omitted | Omitted | Omitted |
| 9 | Not Always | 71 (29.58) | 9 (32.14) | 62 (29.25) | 0.83 | 1 | 1 | 1 |
|  | Always | 169 (70.42) | 19 (67.86) | 150 (70.75) |  | 0.87 (0.29-2.65) | 1.01 (0.31-3.28) | 1.03 (0.32-3.36) |
|  | Q9 Score: Mean (SD) | 0.84 (0.28) | 1 (0) | 0.82 (0.29) | <0.01 | Omitted | Omitted | Omitted |
| 11 | At least once a week | 86 (35.83) | 9 (32.14) | 77 (36.32) | 0.81 | 1 | 1 | 1 |
|  | At least once a day | 150 (62.5) | 19 (67.86) | 131 (61.79) |  | 1.24 (0.41-3.75) | 1.58 (0.49-5.1) | 1.61 (0.50-5.22) |
|  | Others | 4 (1.67) | 0 (0) | 4 (1.89) |  | Omitted | Omitted | Omitted |
|  | Q11 Score: Mean (SD) | 0.74 (0.38) | 0.73 (0.57) | 0.74 (0.38) | 0.94 | 0.96 (0.25-3.73) | 1.38 (0.32-5.90) | 1.42 (0.33-6.11) |
| 12 | Dry wipe | 29 (12.08) | 1 (3.57) | 28 (13.21) | 0.22 | 0.24 (0.02-3.53) | 0.28 (0.03-2.62) | 0.19 (0.01-4.08) |
|  | Wipe with wet cloth | 43 (17.92) | 3 (10.71) | 40 (18.87) | 0.43 | 0.52 (0.1-2.65) | 0.63 (0.11-3.59) | 0.59 (0.1-3.44) |
|  | Cleaned with detergent (soap) | 76 (31.67) | 6 (21.43) | 70 (33.02) | 0.28 | 0.55 (0.16-1.92) | 0.63 (0.17-2.29) | 0.59 (0.16-2.19) |
|  | Cleaned with warm water and detergent (soap) | 155 (64.58) | 18 (64.29) | 137 (64.62) | 1 | 0.99 (0.33-2.91) | 0.80 (0.26-2.49) | 0.79 (0.25-2.46) |
|  | Cleaned with household bleach | 76 (31.67) | 7 (25) | 69 (32.55) | 0.52 | 0.69 (0.21-2.26) | 0.80 (0.23-2.72) | 0.76 (0.22-2.64) |
|  | Others | 23 (9.58) | 4 (14.29) | 19 (8.96) | 0.32 | 1.69 (0.37-7.76) | 1.72 (0.35-8.56) | 1.81 (0.36-9.15) |
|  | Q12 Score: Mean (SD) | 0.34 (0.17) | 0.31 (0.16) | 0.35 (0.17) | 0.32 | 0.28 (0.01-8.42) | 0.33 (0.01-12.15) | 0.26 (0.01-10.41) |
| 12 (Others) | Others (Disinfectant) | 16 (69.57) | 3 (100) | 13 (65) | 0.66 | Omitted | Omitted | Omitted |
|  | Others (Sanitiser) | 5 (21.74) | 0 (0) | 5 (25) |  | Omitted | Omitted | Omitted |
|  | Others (Water) | 0 (0) | 0 (0) | 0 (0) |  | NA | NA | NA |
|  | Others (Alcohol) | 0 (0) | 0 (0) | 0 (0) |  | NA | NA | NA |
|  | Others (Others) | 2 (8.7) | 0 (0) | 2 (10) |  | NA | NA | NA |
| 15 | Yes | 82 (34.17) | 12 (42.86) | 70 (33.02) | 0.30 | 1 | 1 | 1 |
|  | No | 158 (65.83) | 16 (57.14) | 142 (66.98) |  | 0.66 (0.23-1.88) | 0.58 (0.19-1.78) | 0.60 (0.19-1.84) |
|  | Q15 Score: Mean (SD) | 0.88 (0.27) | 0.89 (0.25) | 0.88 (0.28) | 0.76 | 1.24 (0.17-9.25) | 1.03 (0.13-7.96) | 1.03 (0.13-8.09) |
| 15 (Yes) | Allow child to enter centre | 1 (1.43) | 0 (0) | 1 (0.47) | 1 | Omitted | Omitted | Omitted |
|  | Educate and explain to parents potential consequences, deny child entry to centre | 58 (24.17) | 10 (83.33) | 48 (68.57) | 0.49 | 2.29 (0.28-18.76) | 2.75 (0.3-25.07) | 2.45 (0.26-23.17) |
|  | Request for medical endorsement | 72 (30) | 10 (83.3) | 62 (88.59) | 0.64 | 0.65 (0.07-5.93) | 0.57 (0.06-5.63) | 0.50 (0.05-5.14) |
|  | Seek Principal approval | 23 (9.58) | 2 (16.67) | 21 (30) | 0.49 | 0.47 (0.06-3.83) | 0.54 (0.06-4.69) | 0.47 (0.05-4.38) |
|  | Others | 0 (0) | 0 (0) | 0 (0) | NA | NA | NA | NA |
| 17 | Yes | 236 (98.33) | 27 (96.43) | 209 (98.58) | 0.39 | 0.39 (0.02-7.95) | 0.25 (0.01-6.00) | 0.29 (0.01-7.57) |
|  | No | 4 (1.67) | 1 (3.57) | 3 (1.42) |  | 1 | 1 | 1 |
|  | Q17 Score: Mean (SD) | 0.65 (0.23) | 0.62 (0.32) | 0.66 (0.21) | 0.60 | 0.61 (0.10-3.65) | 0.47 (0.07-2.96) | 0.50 (0.07-3.37) |
| 17 (Yes) | Temperature check | 234 (97.5) | 27 (100) | 207 (99.04) | 1 | Omitted | Omitted | Omitted |
|  | Check for relevant symptoms | 236 (98.33) | 27 (100) | 209 (100) | NA | Omitted | Omitted | Omitted |
|  | Others | 17 (7.08) | 1 (3.7) | 16 (7.66) | 0.70 | 0.46 (0.03-6.97) | 0.16 (0.003-9.02) | 0.16 (0.002-9.82) |
| 18 | Sent home | 226 (94.17) | 27 (96.43) | 199 (93.87) | 1 | 1.36 (0.09-21.28) | 2.65 (0.15-47.52) | 2.87 (0.16-50.93) |
|  | Allowed to enter centre but monitored closely | 11 (4.58) | 1 (3.57) | 10 (4.72) |  | 1 | 1 | 1 |
|  | Others | 3 (1.25) | 0 (0) | 3 (1.42) |  | Omitted | Omitted | Omitted |
|  | Q18 Score: Mean (SD) | 0.90 (0.44) | 0.93 (0.38) | 0.89 (0.45) | 0.64 | 1.25 (0.31-5.00) | 1.61 (0.39-6.61) | 1.68 (0.41-6.91) |
| 20 | Isolate child | 216 (90) | 22 (78.57) | 194 (91.51) | 0.04 | 0.34 (0.09-1.31)ᶧ | 0.84 (0.16-4.50) | 0.82 (0.15-4.43) |
|  | Inform parents to pick up child | 232 (96.67) | 25 (89.29) | 2207 (97.64) | 0.05 | 0.20 (0.03-1.43)ᶧ | 0.46 (0.05-3.88) | 0.46 (0.06-3.88) |
|  | Others | 5 (2.08) | 1 (3.57) | 4 (1.89) | 0.47 | 1.93 (0.01-35.98) | 0.08 (0.0002-36.34) | 0.08 (0.0002-35.42) |
|  | Q20 Score: Mean (SD) | 0.46 (0.13) | 0.38 (0.21) | 0.47 (0.11) | 0.04 | **0.03 (0.0008-0.87)*** | NA | NA |
| 20 (Difficulty in getting parents to pick up child) | Yes | 83 (36.24) | 9 (37.5) | 74 (36.1) | 1 | 1 | 1 | 1 |
|  | No | 146 (63.76) | 15 (62.5) | 131 (63.9) |  | 0.94 (0.3-2.97) | 0.93 (0.28-3.12) | 0.93 (0.28-3.12) |

OR, Odds ratio; *p<0.01; ᶧ0.01≤p<0.05; NA, Not Applicable; NE, Not Estimable; p, Fisher’s exact test p-value (categorical variable) or t-test p-value (continuous variable)
All **bolded** odds ratios indicate statistically significance results i.e. p<0.01
^a^ Parents: adjusted for ethnicity, Q16 Score, Q17 Score; Teachers: adjusted for Q6 Score, Q20 Score
^b^ Parents: adjusted for ethnicity, Q16 Score, Q17 Score, HFMD Status (Yes as reference group); Teachers: adjusted for Q6 Score, Q20 Score, HFMD-related centre closure experience (Yes as reference group)
