# Supplementary material for: The knowledge, attitudes and practices of hand, foot, and mouth disease prevention strategies amongst parents and educators of children under 5 years amidst COVID-19 pandemic: A cross-sectional study
Source: Front Public Health. 2022 Oct 17;10:908004. doi: 10.3389/fpubh.2022.908004 (PMC9619192; doi:10.3389/fpubh.2022.908004)
Supplement: Supplementary file 4 [file Data_Sheet_1.PDF]

Parents Survey

Record ID ______________________

Dear Parent,

Invitation to participate in HFMD study by the Centre for Infectious Disease Epidemiology and Research
My name is Nicholas Lau, and I am a research assistant with the Centre for Infectious Disease Epidemiology and Research (CIDER) based in the Saw Swee Hock School of Public Health under the National University of Singapore. On behalf of Assistant Professor Vincent Pang, I would like to invite you to participate in a study on the effectiveness of current strategies used to control the spread of Hand, Foot and Mouth Disease (HFMD) in Child Care Centres (CCCs). In particular, we are interested in finding out what challenges CCCs and parents whose children attend CCCs face in dealing with HFMD cases.
HFMD is an infectious disease that largely affects children aged 5 and below, and its spread is compounded in areas where a large number of children gather. This is why we have chosen to conduct our study in CCCs, in hopes of improving current control strategies and/or coming up with more targeted approaches to minimise the spread of HFMD.
Furthermore, having to care for a child with HFMD can be a distressing period for parents. It is also distressing for CCCs because of the possibility of a potential HFMD outbreak occurring in the centre. Therefore, it is imperative that we work together to step up HFMD control measures to ensure a safe environment for the children to play, learn and grow.
Therefore, we would like to request for your participation in this study. You will be required to complete a survey that will take 10-15 minutes. You can do this by filling in the survey here. If you have more than one child, please only complete the survey once.
Data collection will carry on for a period of 1 month, after which the data will be analysed. We hope to get as many participants as possible to ensure the quality of the study. More information can be found in the study information sheet below. Should you have any other enquiries about the study, please do not hesitate to email me at nicholaslau95@u.nus.edu.
Thank you for taking the time to consider our request. We look forward to hearing from you.

Yours sincerely,
Nicholas Lau
Research Assistant,
Centre for Infectious Disease Epidemiology and Research
Saw Swee Hock School of Public Health
National University of Singapore

Study Information Sheet
1. Protocol title
Barriers to existing Hand, Foot and Mouth Disease containment strategies in Child Care Centres
2. Principal Investigator and co-investigator(s), if any, with the contact number and organization:
Principal Investigator: A/P Pang Junxiong Vincent (ephpjv@nus.edu.sg), Director, Centre for Infectious Disease Epidemiology and Research, NUS Saw Swee Hock School of Public Health (CIDER, NUS SSHSPH)
Co-investigator: Lau Wai Hong Nicholas (nicholaslau95@u.nus.edu), Research Assistant, CIDER, NUS SSHSPH
3. What is the purpose of this research?
You are invited to participate in a research study. This information sheet provides you with information about the research study. The Principal Investigator (the person in charge of this research) or his/her representative will also describe this research to you and answer all of your questions. Read the information below and ask questions about anything you don’t understand before deciding whether or not to take part.
This study aims to assess the effectiveness of current strategies used to control the spread of Hand, Foot and Mouth Disease (HFMD) in Child Care Centres (CCC). A large proportion of patients with HFMD are children aged 5 and below. The spread of the disease is also compounded in areas where a large number of children gather. Therefore, it is in our interest to ensure that the CCCs provide a safe space for the children to learn, play and grow, with minimal risk of getting infected. Through assessing the effectiveness of current strategies, it is hoped that we are able to identify the gaps in current methods so as to come up with more targeted strategies to prevent the spread of HFMD.
4. Who can participate in the research? What is the expected duration of my participation? What is the duration of this research?
All parents whose child(ren) attend a Child Care Centre can take part. Participants will be required to answer a 21-item questionnaire, which will take approximately 10-15 minutes. The study is expected to last for 1 month.
5. What is the approximate number of research participants involved?
The target number of participants for this research study is 1500.
6. What will be done if I take part in this research study?
You will be required to answer an anonymous 21-item questionnaire which will take approximately 10-15 minutes.
7. How will my privacy and the confidentiality of my research records be protected?
Your personal data will not be collected for this study. You may choose to withdraw from the research at any point in time by exiting the e-survey. However, since all responses collected are anonymous, we will be unable to exclude your responses from analysis once the survey has been submitted.
All data collected will be kept in accordance to the University’s Research Data Management Policy. Research data used in any publication will be kept for a minimum of 10 years before being discarded.
8. What are the possible discomforts and risks for participants?
There are no anticipated discomforts or risks for participants.
9. What is the compensation for any injury?
No injury is expected over the course of this research study. Hence, no compensation will be provided.
10. Will there be reimbursement for participation?
No reimbursement will be provided for participation.
11. What are the possible benefits to me and to others?
There is no direct benefit to you by participating in this research study. The knowledge gained may benefit the public in the future. In particular, it will help in our assessment of the current situation of the spread of HFMD in CCCs, which may possibly lead to the implementation of better HFMD control strategies.
12. Can I refuse to participate in this research?
Yes, you can. Your decision to participate in this research study is voluntary and completely up to you. You can also withdraw from the research at any time without giving any reasons, by exiting the e-survey.
13. Whom should I call if I have any questions or problems?
Please contact the Principal Investigator, Pang Junxiong Vincent (ephpjv@nus.edu.sg) or (Attn: Lau Wai Hong Nicholas at email nicholaslau95@u.nus.edu) for all research-related matters and in the event of research-related injuries.
For an independent opinion regarding the research and the rights of research participants, you may contact a staff member of the National University of Singapore Institutional Review Board (Attn: Dr Chan Tuck Wai, at telephone (+65) 6516 1234 or email at irb@nus.edu.sg)

1 Demographic Information[illegible]

Sex[illegible] :	◯ Male[illegible]<br>◯ Female[illegible]
Age[illegible]	__________________________________
Race[illegible]	◯ Chinese[illegible]<br>◯ Malay[illegible]<br>◯ Indian[illegible]<br>◯ Others[illegible]
Others[illegible] :	__________________________________
Occupation[illegible]:	__________________________________
Highest educational qualification:	◯ Pre-primary<br>◯ Primary<br>◯ Secondary<br>◯ Post-Secondary (Non-Tertiary)<br>◯ Diploma courses<br>◯ University<br>◯ Postgraduate<br>◯ Postdoctoral
Housing Type[illegible]:	__________________________________
Type of Child Care Centre your child attends<br>[illegible]	◯ Public[illegible]<br>◯ Private[illegible]<br>◯ Autonomous[illegible]
Number of children[illegible]:	__________________________________
Age(s) of child(ren) [illegible]:	____
Has/have your child(ren) gotten HFMD before?<br>[illegible]	◯ Yes<br>◯ No
If yes, at what age(s)?<br>[illegible]<br>Child 1[illegible]: __________<br>Child 2 [illegible]: __________<br>Child 3 [illegible]: __________	____

2 Survey Questions

Section A. Please answer the following questions based on your own knowledge and understanding of hand, foot and mouth disease (HFMD) without external assistance.

1. HFMD is a ____________ disease.
□□□□□□ ______ □□□□

◯ bacterial □□
◯ viral □□
◯ parasitic □□
◯ fungal □□
◯ I don't know □□□□

2. HFMD can be transmitted via
□□□□□□□ ______ □□□□□
(you may select more than one option
□□□□□□□□□□)

☐ saliva □□
☐ stool □□
☐ fluid from an infected person's blisters □□□□□□□□□
☐ respiratory droplets □□ (e.g. when a person sneezes or coughs □□□□□□□□□□□□□)
☐ touching something that has been touched by someone with HFMD □□□□□□□□□□□
☐ I don't know □□□□

3. The following disinfectant(s) is/are able to kill HFMD-causing agents:
□□□□□□□□□□□□□□□□□□□□
(you may select more than one option□□□□□□□□□□)

☐ alcohol-based sanitisers □□□□□
☐ regular liquid soap □□□□□□
☐ antibacterial liquid soap □□□□□
☐ chlorhexidine (hospital grade soap) □□□□□
☐ household bleach (5% sodium hypochlorite) □□□□□
☐ I don't know □□□□

4. The following is/are possible outcome(s) of HFMD:
□□□□□□□□□□□□
(you may select more than one option□□□□□□□□□□)

☐ Fever □□
☐ Blisters on the hands and feet □□□□□
☐ Mouth ulcers □□□□
☐ Inflammation of the brain□□ (encephalitis)
☐ Inflammation of the heart muscle□□□ (myocarditis)
☐ Inflammation of the airways□□ (pneumonia)
☐ Death □□

5. "The chances of getting infected with HFMD the second time are very slim." Is this statement true or false?
"□□□□□□□□□□□□□□□□□□"□□□□□□□□□□□□□□

◯ True □□
◯ False □□
◯ I don't know □□□□

6. People with HFMD are infectious only when they have symptoms. Once the symptoms go away, they can no longer pass the infection on to other people." Is this statement true or false?
"□□□" □□□□□□□□□□□□□□

◯ True □□
◯ False □□
◯ I don't know □□□□

The following sections are about the challenges you face with regards to caring for the children who attend the Child Care Centre you work at. Please be assured that your responses are 100% anonymous and will not be traceable to your identity. As such, we appreciate your honest responses which will help us to provide better support for Child Care Centres.

Section B.
The following picture is a sample of the “Hand-Washing Technique” Guide. Please answer the following questions truthfully based on your own experiences. [illegible]“[illegible]”[illegible]

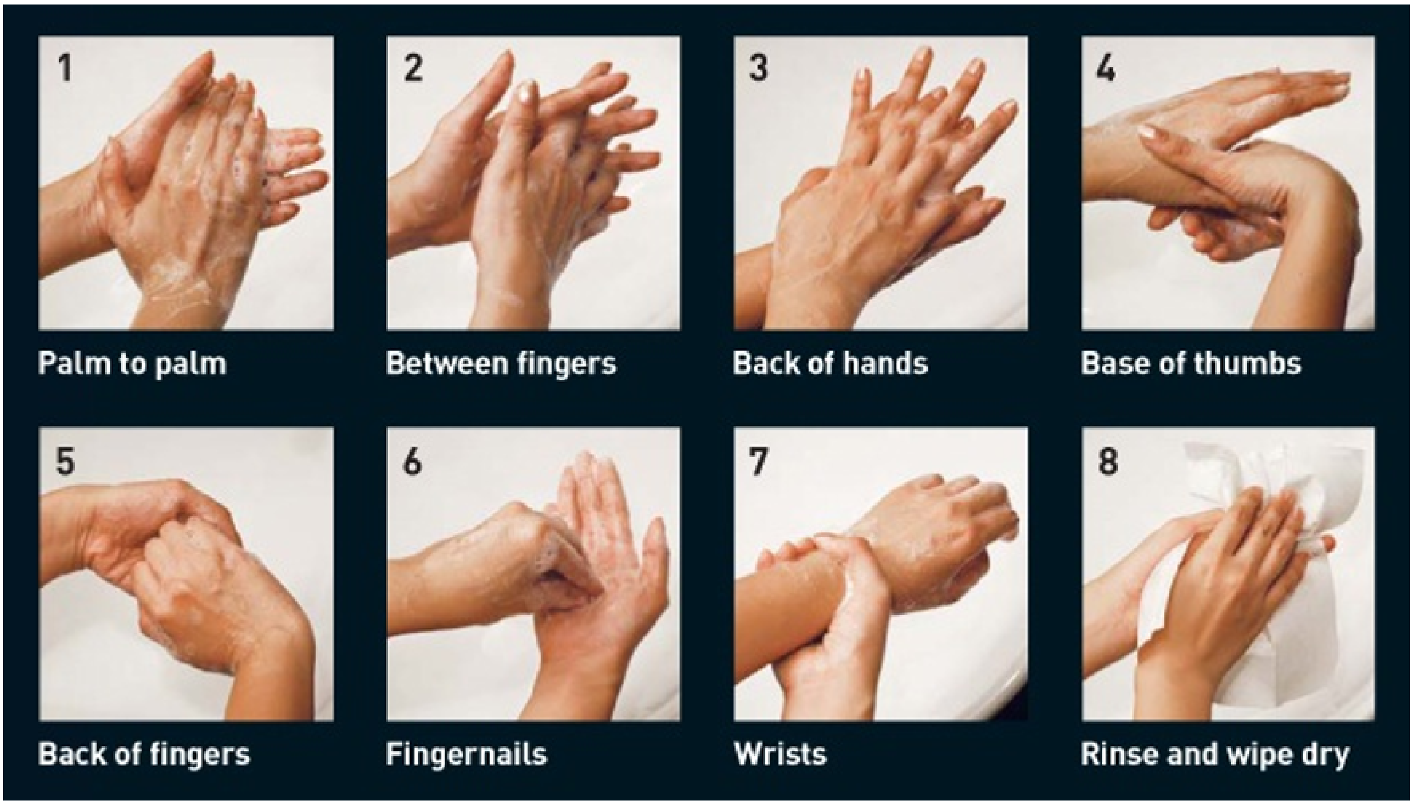

7. I perform all the steps stated in the "Hand-Washing Technique" Guide. [illegible]"[illegible]"[illegible]	◯ Never [illegible]<br>◯ Sometimes [illegible]<br>◯ Always [illegible]<br>◯ When I am prompted by the guide [illegible]"[illegible]"[illegible]
8. When I assist my child(ren) in washing their hands, I make sure that they follow all the steps in the guide. [illegible]"[illegible]"[illegible]	◯ Never [illegible]<br>◯ Sometimes [illegible]<br>◯ Always [illegible]<br>◯ When I am prompted by the guide [illegible]"[illegible]"[illegible]
9. How frequently do you clean your child(ren)'s toys? [illegible]	◯ Everyday [illegible]<br>◯ 3-5 times a week [illegible]3-5 [illegible]<br>◯ Once a week [illegible]<br>◯ Once every 2 weeks [illegible]<br>◯ Once a month [illegible]<br>◯ Less than once a month [illegible]<br>◯ Never [illegible]
10. When do you clean your child(ren)'s toys? [illegible]<br>to correspond responses with that for qns 9 for logic	◯ Before and after play time [illegible]<br>◯ Only before play time [illegible]<br>◯ Only after play time [illegible]<br>◯ Never [illegible]<br>◯ Others [illegible]:
Others	______________________________

10. Getting the children to wash their hands using the 7-step Hand-Washing Technique every time is (you may select more than one option)

- ◯ a. time-consuming
- ◯ b. important
- ◯ c. tedious
- ◯ d. excessive
- ◯ e. protective against infectious diseases
- ◯ f. others

11. What are your child(ren)'s toys cleaned with? [illegible]
(You may select more than one option[illegible])

to correspond responses with that for qns 9/10 for logic

- ☐ They are dry wiped [illegible]
- ☐ They are wiped with a wet cloth [illegible]
- ☐ They are cleaned with detergent (soap) [illegible]
- ☐ They are cleaned with warm water and detergent (soap) [illegible]
- ☐ They are cleaned with household bleach[illegible] (5% sodium hypochlorite)
- ☐ They are not cleaned [illegible]
- ☐ Others [illegible]:

Others ______________________________

12. Which of the following do you use to clean the house?
[illegible]
(You may select more than one option[illegible])

- ◯ It is dry wiped[illegible]
- ◯ It is cleaned with a wet mop or cloth [illegible]
- ◯ It is cleaned with detergent (soap) [illegible]
- ◯ It is cleaned with warm water and detergent (soap) [illegible]
- ◯ It is cleaned with household bleach[illegible] (5% sodium hypochlorite)
- ◯ It is not cleaned [illegible]
- ◯ Others [illegible]:

Others ______________________________

13. How often do you use household bleach (5% sodium hypochlorite)?
[illegible]

to correspond responses with that for qns 11,12 for logic

- ◯ Never [illegible]
- ◯ Once a month [illegible]
- ◯ Once every other week [illegible]
- ◯ 1-2 times per week [illegible]1-2 [illegible]
- ◯ 3-5 times per week [illegible]3-5 [illegible]
- ◯ Everyday [illegible]
- ◯ When someone in the household is sick [illegible]
- ◯ Others [illegible]:

Others ______________________________

14. Typically, doctors issue a Medical Certificate (MC) for those diagnosed with HFMD. This would mean that the infected person is exempted from school or work for the duration of the MC. In the event that your child is diagnosed with HFMD and is given an MC, which of the following would you do? You may select more than one option.
[illegible](MC)[illegible]MC[illegible]MC[illegible]

☐ Inform the Child Care Centre immediately
[illegible]
☐ Keep them at home until the MC expires
[illegible]MC[illegible]
☐ Keep them at home until all the symptoms disappear, even if it is shorter than the duration of the MC
[illegible]MC[illegible]
☐ Keep them at home until all the symptoms disappear, even if it is longer than the duration of the

[illegible]MC[illegible]

☐ Bring them back to the Child Care Centre once they feel better, even if symptoms still persist

[illegible]

Section D.

Please answer the following questions truthfully based on your thoughts and feelings about HFMD. [illegible]

15. Washing my hands using the 7-step "Hand-Washing Technique" every time is [illegible]"[illegible]"[illegible]
(you may select more than one option[illegible])

☐ time-consuming [illegible]
☐ important [illegible]
☐ tedious [illegible]
☐ excessive [illegible]
☐ protective against infectious diseases [illegible]
☐ others [illegible]:

Others ______________________________

16. Getting my children to wash their hands using the 7-step "Hand-Washing Technique" every time is [illegible]"[illegible]" [illegible]
(you may select more than one option[illegible])

☐ time-consuming [illegible]
☐ important [illegible]
☐ tedious [illegible]
☐ excessive [illegible]
☐ protective against infectious diseases [illegible]
☐ others [illegible]:

Others ______________________________

17. Disinfecting my child(ren)'s toys regularly is [illegible]
(you may select more than one option[illegible])

☐ time-consuming [illegible]
☐ important [illegible]
☐ tedious [illegible]
☐ excessive [illegible]
☐ protective against infectious diseases [illegible]
☐ others [illegible]:

Others ______________________________

18. Cleaning and disinfecting frequently touched surfaces (floor, tables, low shelves and doorknobs) regularly is [illegible]
(you may select more than one option[illegible])

☐ time-consuming [illegible]
☐ important [illegible]
☐ tedious [illegible]
☐ excessive [illegible]
☐ protective against infectious diseases [illegible]
☐ others [illegible]:

Others ______________________________

19. In the event of an outbreak of HFMD in a school or Child Care Centre, the Ministry of Health might order that the school or centre to close down for a duration of approximately 10 days. Select all the options that apply to you. [illegible]

a. I feel that 10 days is [illegible]

☐ reasonable [illegible]
☐ too long [illegible]
☐ too short [illegible]

---

b. I feel that short-term closure (at least 10 days) of the centre or school [illegible]

☐ can help to stop the spread of HFMD [illegible]
☐ is not necessary [illegible]
☐ will be too much of a burden to me [illegible] Please elaborate (e.g., the need to take leave, no alternate child care arrangement, etc.) [illegible] :
☐ poses inconvenience to me, but I will be able to make the appropriate arrangements to cope

[illegible]
☐ others [illegible]:

---

will be too much of a burden to me
[illegible]
Please elaborate (e.g., the need to take leave, no alternate child care arrangement, etc.)
[illegible] :

____

____

Others

____

---

20. Currently, there are no approved vaccinations for HFMD in Singapore. However, in the event that it becomes available in Singapore, would you bring your child(ren) for vaccination against HFMD?

○ Yes [illegible]
○ No [illegible]
○ Depends

Why? [illegible]

____

If you answered "depends", what will you consider when deciding whether to bring your child(ren) for vaccination against HFMD? You may select more than one option[illegible].

☐ Cost [illegible]
☐ Safety
☐ Whether is is covered by insurance
☐ The number of doses required
☐ How effective the vaccine is
☐ Doctor's recommendation
☐ The age of my child
☐ The risk of my child getting infected with HFMD
☐ The risk of my child getting reinfected with HFMD
☐ Others:

Others

____

---

21. I would like to know more about HFMD.

○ Yes [illegible]
○ No [illegible]

If yes, which type(s) of media would you prefer?

☐ Brochure
☐ Talk by a healthcare professional
☐ Poster
☐ Social media posts (e.g. Facebook, Instagram, Youtube, etc.)
☐ Others [illegible]:

Others

____

Thank you for finishing the survey
