# Supplementary material for: The knowledge, attitudes and practices of hand, foot, and mouth disease prevention strategies amongst parents and educators of children under 5 years amidst COVID-19 pandemic: A cross-sectional study
Source: Front Public Health. 2022 Oct 17;10:908004. doi: 10.3389/fpubh.2022.908004 (PMC9619192; doi:10.3389/fpubh.2022.908004)
Supplement: Supplementary file 5 [file Data_Sheet_2.PDF]

Teacher's Survey

Record ID	__________________________________
Sex :	◯ Male<br>◯ Female
Age in Numbers	__________________________________
Race	◯ Chinese<br>◯ Malay<br>◯ Indian<br>◯ Others
Specify your race:	__________________________________
Highest educational qualification:	◯ Pre-primary<br>◯ Primary<br>◯ Secondary<br>◯ Post-Secondary (Non-Tertiary)<br>◯ Diploma courses<br>◯ University<br>◯ Postgraduate<br>◯ Postdoctoral
Number of years worked in a CCC:	__________________________________
Are you a full-time or part-time staff?	◯ Full-time<br>◯ Part-time
Type of CCC you work at	◯ Private<br>◯ Public<br>◯ Autonomous
What is the typical staff to children ratio? 1 staff : __ children	__________________________________
Do you have children?	◯ Yes<br>◯ No
If yes, how many?	__________________________________
If yes, do you send them to a CCC?	◯ Yes<br>◯ No
1. Hand, foot and mouth disease (HFMD) is a ________ disease.	◯ a. bacterial<br>◯ b. viral<br>◯ c. fungal<br>◯ d. parasitic<br>◯ e. I don't know

2. HFMD can be transmitted via (you may select more than one option)	☐ a. saliva<br>☐ b. stool (faeces)<br>☐ c. fluid from the blisters of someone with HFMD<br>☐ d. respiratory droplets (e.g. when a person coughs or sneezes)<br>☐ e. touching something that has been touched by someone with HFMD<br>☐ f. I don't know
3. The following disinfectant(s) is/are able to kill HFMD-causing agents:(you may select more than one option)	☐ a. Alcohol-based sanitiser<br>☐ b. Regular liquid soap<br>☐ c. Antibacterial liquid soap<br>☐ d. Chlorhexidine (hospital grade soap)<br>☐ e. Household bleach (5% sodium hypochlorite)<br>☐ f. I don't know
4. The following are symptoms of HFMD: (you may select more than one option)	☐ a. Fever<br>☐ b. Blisters on the hands and feet<br>☐ c. Ulcers in the mouth<br>☐ d. Poor appetite<br>☐ e. Vomitting<br>☐ f. Lethargy<br>☐ g. I don't know
5. "The chances of getting infected with HFMD the second time are very slim." Is this statement true or false?	◯ a. True<br>◯ b. False<br>◯ c. I don't know
6. "People with HFMD are infectious only when they have symptoms. Once the symptoms go away, they can no longer pass the infection on to other people." Is this statement true or false?	◯ a. True<br>◯ b. False<br>◯ c. I don't know

The following sections are about the challenges you face with regards to caring for the children who attend the Child Care Centre you work at. Please be assured that your responses are 100% anonymous and will not be traceable to your identity. As such, we appreciate your honest responses which will help us to provide better support for Child Care Centres.

Hand-washing

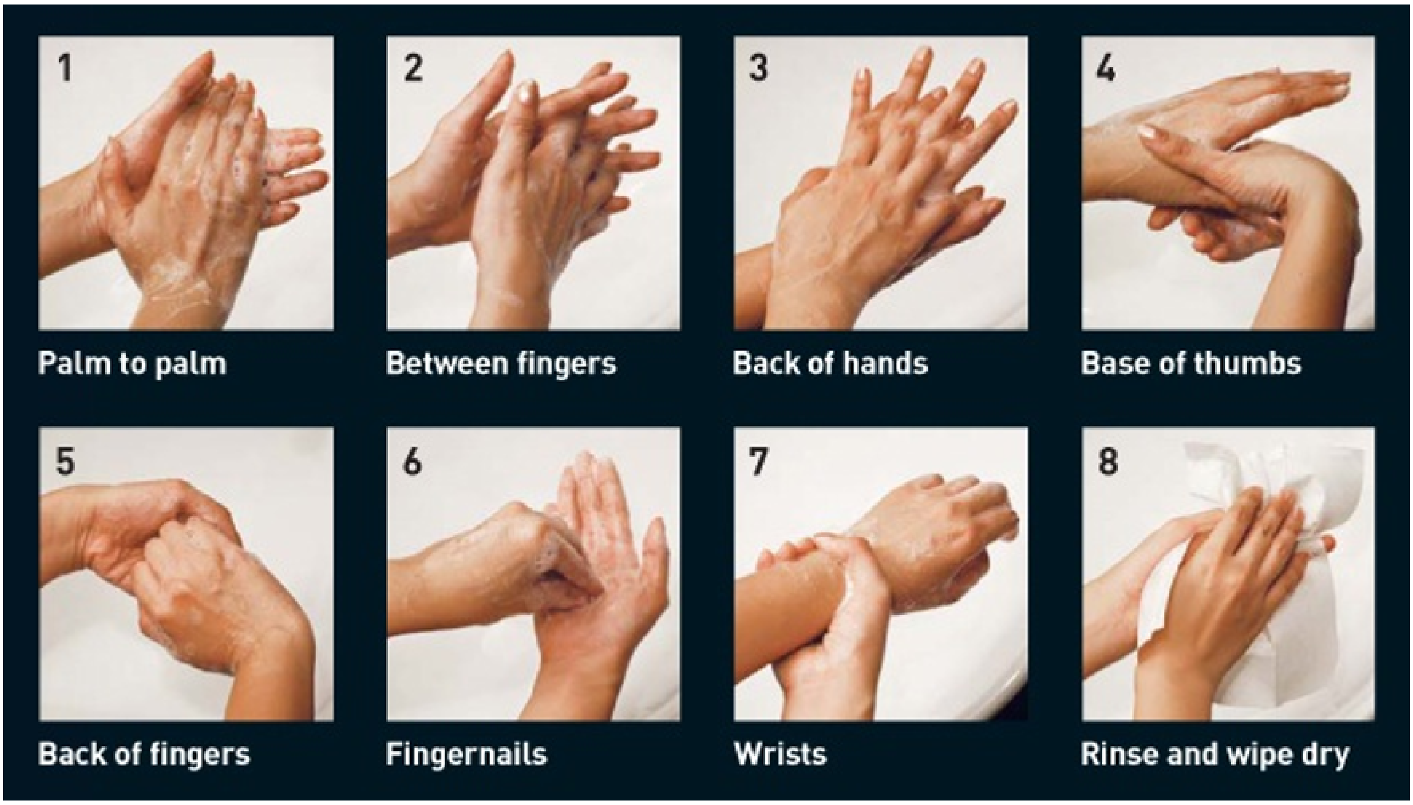

7. I follow the steps given in "Hand-Washing Technique" guide.

- ◯ a. Always
- ◯ b. Sometimes
- ◯ c. Never
- ◯ d. When I am prompted by the guide

8. Washing my hands using the 7-step Hand-Washing Technique every time is (you may select more than one option)

- ☐ a. time-consuming
- ☐ b. important
- ☐ c. tedious
- ☐ d. excessive
- ☐ e. protective against infectious diseases
- ☐ f. others

Others

____

9. When I assist the children in washing their hands, I make sure that the children follow all the steps stated in the guide.

- ◯ a. Always
- ◯ b. Sometimes
- ◯ c. Never
- ◯ d. When I am prompted by the guide

10. Getting the children to wash their hands using the 7-step Hand-Washing Technique every time is (you may select more than one option)

- ◯ a. time-consuming
- ◯ b. important
- ◯ c. tedious
- ◯ d. excessive
- ◯ e. protective against infectious diseases
- ◯ f. others

Others

____

Cleaning Practices

11. How frequently are the toys in the centre cleaned?	◯ a. More than once a day<br>◯ b. Once a day<br>◯ c. 2-3 times a week<br>◯ d. Once a week<br>◯ e. Others
Others	__________________
12. What are the toys cleaned with? (you may select more than one option)	☐ a. They are dry-wiped<br>☐ b. They are wiped with a wet cloth<br>☐ c. They are cleaned with detergent (soap)<br>☐ d. They are cleaned with warm water and detergent (soap)<br>☐ e. They are cleaned with household bleach (5% sodium hypochlorite)<br>☐ f. Others:
Others	__________________
13. Disinfecting the child(ren)'s toys regularly is (you may select more than one option)	☐ a. time-consuming<br>☐ b. important<br>☐ c. tedious<br>☐ d. excessive<br>☐ e. protective against infectious diseases<br>☐ f. others
Others	__________________
14. Cleaning and disinfecting frequently touched surfaces (floor, tables, low shelves and doorknobs) regularly is (you may select more than one option)	☐ a. time-consuming<br>☐ b. important<br>☐ c. tedious<br>☐ d. excessive<br>☐ e. protective against infectious diseases<br>☐ f. others
Others	__________________
Medical Certificate	
15. Occasionally, children fall ill and are given a medical certificate (MC) by the doctor requiring them to stay home for a period of time. Have you encountered parents who don't observe the full duration of the MC and still bring their child to the centre?	◯ Yes<br>◯ No
If yes, what did you do? (you may select more than one option)	☐ a. Allow the child to enter the centre<br>☐ b. Educate and explain to the parents the potential consequences and deny the child entry<br>☐ c. Request for the parents to seek medical endorsement that the child is well and fit to return to the centre<br>☐ d. Seek the Principal for approval<br>☐ e. Others

Others

____

16. What are some of some of the reasons that parents have given for not observing the full duration of the MC? (Please answer "NA" if not applicable)
to correspond responses with qns 15 for logic

____

Health Check

17. Is there a health check routine for when the children are first brought to the centre in the day?

◯ Yes
◯ No

If yes, what is done during the health check? Please select all that apply.

☐ a. Temperature check
☐ b. Check for unusual rashes, blisters or ulcers on the hands and feet, and in the mouth
☐ c. Others:

Others

____

18. What happens if a child fails the health check?
to correspond responses with that for qns 17 for logic

◯ a. They are sent home
◯ b. They are allowed to enter the centre but will be monitored closely
◯ c. Others:

Others

____

19. Have you had difficulties getting parents to comply with the centre's decision?

◯ Yes
◯ No

If yes, what were some of the reasons given by parents to not comply?
to correspond responses with that for qns 19 for logic

____

During the day

20. In the event that the child starts to show symptoms during the day (e.g. he/she develops a fever), what do you do? Please select all that apply.

to correspond responses with that for qns 17 for logic

☐ a. Isolate the child at the sick bay or a separate classroom
☐ b. Call to inform the parents, and ask them to come and pick their child
☐ c. Others:

Others

____

If you answered "b. Call to inform the parents, and ask them to come and pick their child", have you had difficulties getting parents to comply with this?
to correspond responses with that for qns 20 for logic

◯ Yes
◯ No

If yes, what were some of the reasons given by parents?

____

School Closure
In the event of an outbreak of HFMD in a school or Child Care Centre, the Ministry of Health might order that the school or centre to close down for a duration of approximately 10 days. Select all the options that apply to you.

21. I feel that 10 days is

◯ a. Reasonable
◯ b. Too long
◯ c. Too short

22. Has the centre you work at been asked to close because of an HFMD outbreak?

◯ Yes
◯ No

If yes, please answer the next question based on your experience.
If no, please answer the next question based on what you think you might experience.

23. What were some of the difficulties you faced when the centre was closed?

____

24. Any suggestions to improve this policy? (Please answer "NA" if not applicable)

____

Others

25. Have you encountered any other challenges when dealing with HFMD cases in the centre that were not mentioned above? What are they? (Please answer "NA" if not applicable)

____

26. Do you have any recommendations for how you can be better supported with regards to dealing with HFMD? (Please answer "NA" if not applicable)

____

27. I would like to know more about HFMD.

◯ Yes ◯ No

If yes, which type(s) of media would you prefer?

◯ a. Brochure
◯ b. Talk by a healthcare professional
◯ c. Poster
◯ d. Social media posts (e.g. Facebook, Instagram, Youtube, etc.)
◯ e. Others:

Others

____

Thank you for finishing the survey
